# Molecular heterogeneity at the network level: high-dimensional testing, clustering and a TCGA case study

**DOI:** 10.1093/bioinformatics/btx322

**Published:** 2017-05-23

**Authors:** Nicolas Städler, Frank Dondelinger, Steven M Hill, Rehan Akbani, Yiling Lu, Gordon B Mills, Sach Mukherjee

**Affiliations:** 1 Global Pricing and Market Access, F. Hoffmann-La Roche Ltd, Basel, Switzerland; 2 Lancaster Medical School, Lancaster University, Lancaster, UK; 3 MRC Biostatistics Unit, University of Cambridge, Cambridge, UK; 4 Department of Bioinformatics and Computational Biology, The University of Texas MD Anderson Cancer Center, Houston, TX, USA; 5 Department of Systems Biology, The University of Texas MD Anderson Cancer Center, Houston, TX, USA; 6 German Centre for Neurodegenerative Diseases (DZNE), Bonn, Germany

## Abstract

**Motivation:**

Molecular pathways and networks play a key role in basic and disease biology. An emerging notion is that networks encoding patterns of molecular interplay may themselves differ between contexts, such as cell type, tissue or disease (sub)type. However, while statistical testing of differences in mean expression levels has been extensively studied, testing of network differences remains challenging. Furthermore, since network differences could provide important and biologically interpretable information to identify molecular subgroups, there is a need to consider the unsupervised task of learning subgroups and networks that define them. This is a nontrivial clustering problem, with neither subgroups nor subgroup-specific networks known at the outset.

**Results:**

We leverage recent ideas from high-dimensional statistics for testing and clustering in the network biology setting. The methods we describe can be applied directly to most continuous molecular measurements and networks do not need to be specified beforehand. We illustrate the ideas and methods in a case study using protein data from The Cancer Genome Atlas (TCGA). This provides evidence that patterns of interplay between signalling proteins differ significantly between cancer types. Furthermore, we show how the proposed approaches can be used to learn subtypes and the molecular networks that define them.

**Availability and implementation:**

As the Bioconductor package nethet.

**Supplementary information:**

Supplementary data are available at *Bioinformatics* online.

## 1 Introduction

Molecular interplay plays a fundamental role in biology and its dysregulation is a feature of many diseases. It is thought that networks encoding molecular interplay may depend on biological context such as cell type, tissue type, or disease subtype. An increasing number of studies, including, among others, ENCODE (Andersson *et al.*, 2014), BLUEPRINT (Martens and Stunnenberg, 2013) and TCGA (The Cancer Genome Atlas Network, 2012), span multiple biological contexts and such studies offer an opportunity to better understand molecular heterogeneity.

The study of molecular networks involves relatively complex statistical models, since it is not only the mean levels of molecular variables but also measures of interplay between them that are relevant. For this reason, despite the importance of networks in biology, several core bioinformatics tasks remain challenging in the network setting. In this paper we address two of these, two-sample testing and clustering. In network models, the number of statistical parameters may grow rapidly with the number of molecular variables and for this reason network-based analyses typically lead to so-called high-dimensional statistical problems, where the number of parameters is large in relation to the sample size.

To fix ideas and clarify the specific questions we address, first consider molecular data $X_{1},X_{2}$ from two groups, each with the same set of variables measured, but with potentially different sample sizes. Using these data, we would like to test significance of differences between the groups, not only in terms of average molecular abundance (as tested in standard differential expression analyses and multiple testing extensions thereof), but at the level of networks describing interplay between the variables. In the general case group-specific networks are not known in advance but must be estimated from the data and the resulting variability in estimation must be properly accounted for. This is the testing problem that we address.

Next consider the unknown groups case, where starting with a dataset X (with no group labels) we seek to identify subsets of samples (i.e. clusters) with their associated networks. In general neither the cluster assignments nor cluster-specific networks are known at the outset. This is the clustering problem that we address.

The testing and clustering problems are different, but share the need to model underlying networks. We use sparse Gaussian graphical models (GGMs) for this purpose. The approaches we discuss are likelihood-based and should be extensible to other classes of model. Estimation for GGMs has been widely studied, including in bioinformatics (e.g. Schäfer and Strimmer, 2005). There has been progress in high-dimensional methods relevant to testing and clustering using GGMs (including Chen and Qin, 2010; Städler and Mukherjee, 2013, 2017; Zhou *et al.*, 2009). We note that GGMs are not causal models and we do not consider causality *per se* in this paper, although extensions in a causal direction could be possible.

We address the testing problem using a framework proposed in Städler and Mukherjee (2017) that extends the likelihood ratio test to the high-dimensional setting. Specifically, we use an application of their methodology to testing networks called *Differential Network* or *DiffNet*. This is a formal statistical test that retains validity in the high-dimensional setting. For a review of related methods and a discussion of methodological differences with respect to the DiffNet test, we refer the interested reader to the reference.

For the clustering problem, we pursue a model-based approach. In particular, we use mixture models where the different mixture components (or clusters) are represented by cluster-specific means and GGMs. Gaussian mixture models have been well-studied in the low-dimensional setting but estimation remains challenging in the high-dimensional setting. In the present context, we are interested in interplay between molecular variables and furthermore we expect that clusters may have different underlying GGMs. This requires formulations for which classical maximum likelihood is ill-suited (even when the number of molecular variables *p* itself is not very large). A computationally and statistically attractive approach is to use $\ell_{1}$-penalization within a mixture model framework and this is the route we pursue. Specifically, we develop a latent variable extension of the graphical lasso (Friedman *et al.*, 2008) that we call *Mixture Graphical Lasso*, or *MixGlasso*. MixGlasso renders estimation tractable by assuming sparsity of the cluster-specific networks, i.e. for each cluster only relatively few edges are important (but these edges are not pre-specified and can differ between clusters).

The features of MixGlasso that make it practically applicable for bioinformatics are: (i) MixGlasso allows clusters to have different means and networks (i.e. GGMs). (ii) The penalty is designed to automatically adapt to the number of clusters and to the sample size and scale of the clusters (these are unknown at the outset and cannot be dealt with by pre-processing). (iii) MixGlasso uses theoretical results from high-dimensional regression to set the level of penalization automatically, following recent work in the context of hidden Markov models (Städler and Mukherjee, 2013). This means that the procedure is essentially free of tuning parameters and efficient enough to be run on a single core.

Many clustering methods that are widely used in bioinformatics, including K-means, PAM and hierarchical clustering, differ fundamentally from MixGlasso in that they are driven by mean levels of variables and do not account for network or covariance structure. The mclust multivariate clustering tool (Fraley *et al.*, 2012) is very popular. The key difference between mclust and MixGlasso is that the former is not geared towards high-dimensional problems. Mukherjee and Hill (2011) discuss penalized, network-based clustering, but using a heuristic algorithm that unlike MixGlasso is not a principled mixture model. The iCluster methodology (Shen *et al.*, 2009) performs high-dimensional clustering via a low-dimensional representation; this differs in intent from MixGlasso, which emphasizes the network setting in which the original molecular variables and their network connections are of direct interest.

Pan and Shen (2007) used penalization for variable selection in clustering, using a mixture model with common diagonal covariance matrices. In contrast, MixGlasso focuses on the non-identical, non-diagonal case that is relevant to discovery of clusters that may have different underlying patterns of molecular interplay. Our approach is similar to Zhou *et al.* (2009) but differs in the form of the penalty: the MixGlasso penalty is designed to automatically adapt to the sample size and scale of clusters and the level of penalization is set automatically.

In summary, the specific contributions of this paper are: (1) We discuss how the DiffNet test can be used for network-related testing in bioinformatics. (2) We propose a penalized mixture model MixGlasso that can be used to cluster data that is likely to be heterogeneous with respect to underlying networks and that automatically takes care of several practical issues; and (3) We illustrate the properties and use of the two approaches by way of simulations and a TCGA case study.

We illustrate the approaches in an analysis of protein data from n=3467 TCGA samples (data from Akbani *et al.*, 2014). Using DiffNet we show that patterns of protein-protein interplay differ significantly between cancer types, both at the ‘global’ level of all assayed proteins and at the ‘local’ level of pre-defined signaling pathways. This offers evidence, over thousands of samples, supporting the notion that signaling depends on disease lineage and context, i.e. that pathways and networks are contextual. Furthermore, using MixGlasso, we identify clusters (that can span more than one cancer type or classical lineage) each having a cluster-specific network. This analysis supports, from a network perspective, the emerging notion that there may be molecular commonalities between seemingly distinct cancer types (see e.g. The Cancer Genome Atlas Network, 2012).

## 2 Materials and methods

We first introduce some notation. Let the number of samples be *n*, the number of variables be *p* and the *n *×* p* data matrix be $X={\left[X_{1}\ldots X_{n}\right]}^{T}$. Data subsets or groups are indexed by k∈{1,…,K}. For each group *k*, *n_k_* denotes the (group-specific) sample size and $X_{k}$ is the corresponding $n_{k}\times p$ data matrix. Group-specific mean vectors and inverse covariance matrices are *μ_k_* and Ω_*k*_ respectively.

### 2.1 DiffNet: testing differences in patterns of molecular interplay

To test whether known groups differ with respect to molecular networks, a starting point is to learn a network model for each group and to then compare the models. Although many procedures are available for learning networks (see e.g. De Smet and Marchal, 2010), the models are inherently complex and typically subject to high statistical variability. This means that observed differences between fitted models may simply be due to such variability. This motivates a need for uncertainty quantification.

The DiffNet test that we use is based on a framework that extends the likelihood ratio test (LRT) to high-dimensions (Städler and Mukherjee, 2017). DiffNet assumes that the data are generated from GGMs and tests the null hypothesis that both groups share the same underlying model, i.e. the null hypotheses

(2.1) $$H_{0}^{\left(k,k^{\prime }\right)}:\Omega_{k}=\Omega_{k\prime },\;k,k\prime \in \{1,\ldots ,K\}\;\;\text{and}\;\;k\neq k\prime .$$

The key idea in DiffNet is to exploit estimated sparsity patterns in the construction of the test statistic and in *P*-value calculation. The use of sparse structure renders the test effective in high-dimensions but raises technical questions that are addressed via theory that extends the LRT to the high-dimensional setting. This gives an asymptotic *P*-value that remains valid in high-dimensional problems.

DiffNet uses randomized data-splitting: sparsity structure is estimated using the first half of the data, and *P*-value calculation carried out using the second half. We consider two variants of DiffNet, one using a single data split (‘DiffNet(SS)’) and one using multiple data splits (50 data splits) followed by *P*-value aggregation (‘DiffNet(MS)’). For full technical details we refer the interested reader to Städler and Mukherjee (2017). The overall analysis is computationally tractable: on the TCGA protein data discussed below a typical (single-split) run of DiffNet required 2.5 minutes and 2.5 GB of memory (on one core of an Intel Nehalem processor).

### 2.2 Subtype identification using MixGlasso

MixGlasso is a penalized mixture of Gaussian graphical models. As above, let *k* index groups and *K* denote the number of groups (in the clustering setting both *K* and cluster assignments are unknown at the outset). Let $S_{i}\in \{1,\ldots ,K\}$ be latent labels with $S_{i}=k$ if sample *i* belongs to group *k*. The component probabilities (i.e. mixture weights) are $\pi_{k}=P(S_{i}=k)$. We assume $X_{i}|S_{i}=k∼N(\mu_{k},\Omega_{k}^{-1})$, where N(μ,Σ) denotes a normal density with mean *μ* and covariance matrix Σ. The mixture model is then parameterized by $\Theta_{K}=(\theta_{1},\ldots ,\theta_{K},\pi_{1},\ldots ,\pi_{K}),\;\theta_{k}=(\mu_{k},\Omega_{k})$, with log-likelihood

(2.2) $$\ell (\Theta_{K};X)=\sum_{i}\;\text{log}\;\left(\sum_{k}\pi_{k}\;N(X_{i}|\mu_{k},\Omega_{k}^{-1})\right).$$

Estimation comprises two (coupled) tasks. The first task is to estimate Θ_*K*_, given the number of clusters *K*. In MixGlasso this is done by minimizing the negative penalized log-likelihood to get

(2.3) $${\hat{\Theta }}_{K,\lambda }=\underset{\Theta_{K}}{\arg\min}-\ell (\Theta_{K};X)+\lambda \;\text{pen}(\Theta_{K}),$$

where

$$\text{pen}(\Theta_{K})=\sum_{k=1}^{K}\pi_{k}^{1/2}\sum_{j\neq j\prime }|\Omega_{k;jj\prime }|/\sqrt{\Omega_{k;jj}\Omega_{k;j\prime j\prime }}$$

is the penalty function and *λ* is a regularization parameter. This specific form of penalty, originally introduced in Städler and Mukherjee (2013) for hidden Markov models, adapts to the sample size and scale of individual clusters. Optimization of (2.3) is performed using expectation-maximization (EM) as outlined in SI.

We set *λ* to a ‘universal’ value $\lambda_{\text{uni}}=\sqrt{2n\;\text{log}\;p}/2$. This penalty is based on well-known theoretical results for high-dimensional regression and the connection between GGMs and regression, and in the present setting is valid for the specific penalty given above (for details see Städler and Mukherjee, 2013). The use of $\lambda_{\text{uni}}$ coupled with the penalty above in effect allows automatic adaptation to cluster size and scale during the EM. As we show below, this allows MixGlasso to give good results across a range of settings, while controlling the computational burden; running MixGlasso on the TCGA dataset (see below) with K=9 clusters required 2 minutes and 5.3 GB of memory (on one core of an Intel Nehalem processor).

The second task involves determining an appropriate number of clusters $K^{*}$. This is done by minimizing the BIC score (*λ* is set to $\lambda_{\text{uni}}$):

$$K^{*}=\underset{K}{\arg\min}\text{BIC}({\hat{\Theta }}_{K,\lambda_{\text{uni}}}),$$

with

$$\begin{array}{l} \text{BIC}({\hat{\Theta }}_{K,\lambda_{\text{uni}}})=-\ell ({\hat{\Theta }}_{K,\lambda_{\text{uni}}};X)+\frac{1}{2}\text{log}\;(n)(K-1)\\ +\frac{1}{2}\text{log}\;(n)\sum_{k=1}^{K}\text{Df}(k,\lambda_{\text{uni}}), \end{array}$$

where degrees of freedom are set as $\text{Df}(k,\lambda_{\text{uni}})=p+\sum_{l\prime \geq l}1_{{\left({\hat{\Omega }}_{k,\lambda_{\text{uni}}}\right)}_{ll\prime }\neq 0}\;$.

## 3 Results

### 3.1 Testing differences in patterns of molecular interplay

**Fig. 1.**
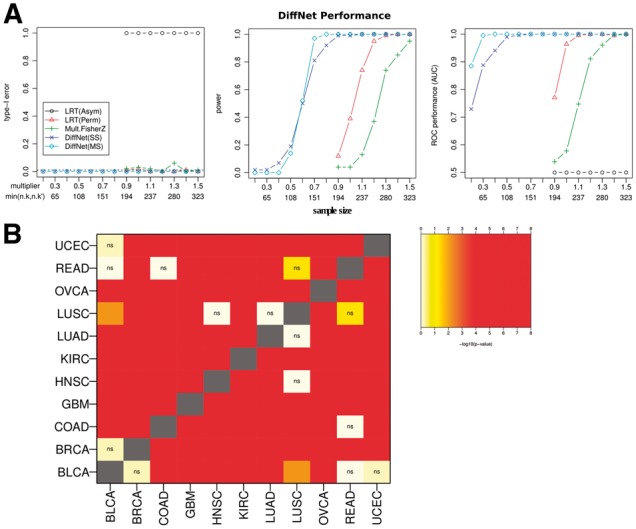
(**A**) Differential network (DiffNet), simulation study. Left-to-right (all as a function of sample size): Type-I error (false positive rate); power (true positive rate); and area under the ROC curve. Average performance over 100 simulation runs shown. The simulation was designed to mimic characteristics and sample size of the TCGA protein data for LUAD and GBM (see SI for details). *X*-axis shows the smaller of the sample sizes of the two compared groups and the multiplier with respect to the TCGA sample sizes. [‘LRT(Asym)’: asymptotic likelihood ratio test (LRT); ‘LRT(Perm)’: permutation-based LRT; ‘Mult.FisherZ’: Fisher’s Z Transform; ‘DiffNet(SS)’: single-split DiffNet; ‘DiffNet(MS)’: multi-split DiffNet (see text)]. (**B**) DiffNet results, TCGA protein data. Heatmap shows FDR-corrected *P*-values indicating significance of differences between 11 cancer types, using data for 181 proteins, including post-translational modifications (see Methods). To focus on network-related differences rather than differential expression, data from each cancer type were normalized to have zero mean and unit variance as a pre-processing step. [ns indicates non-significant cancer pairs with FDR > 1%] (Color version of this figure is available at *Bioinformatics* online.)

**Simulation study.** Figure 1A presents results of a simulation study, based on the characteristics of the TCGA data (including sample size and dimensionality; see SI for details), that compares differential network (‘DiffNet’) against standard multivariate tests. The methods we compare against are:

1. *Likelihood ratio test (‘LRT (Asym)’)*. This is a classical LRT, based on the Gaussian models and with an asymptotic *P*-value.
2. *Permutation LRT (‘LRT(Perm)’)*. This uses the same test statistic as the classical LRT, but obtains a *P*-value by permutation of group labels.
3. *Test based on Fisher’s Z-transform (‘Mult.FisherZ’)*. Here, equality of all partial correlations is tested using *P*-values obtained by transforming each partial correlation using Fisher’s Z-transform (see SI Section 2.2).

In line with theoretical results, we find that DiffNet controls type I error. Both DiffNet variants, but especially the multi-split variant, outperform the other approaches in an ROC sense and in terms of power. The permutation LRT is considerably less powerful than DiffNet, but as expected catches up at larger sample sizes. The poor performance of the classical LRT is expected (due to the high-dimensional nature of the problem) and it is interesting to note that LRT(Asym) does not control Type I error even with 50% more samples than available in TCGA (i.e. more than 300 samples per group; with number of variables *p* = 181). Additional simulations using a non-Gaussian model appear in Supplementary Figure S1A.

**TCGA data.** Next, we considered TCGA protein data (spanning p=181 proteins listed in Supplementary Table S1) from Akbani *et al.* (2014). Normalization for batch effects using control samples is discussed in Akbani *et al.* (2014); here, we use the normalized data presented there and refer the interested reader to the reference for details.

Akbani *et al.* (2014) inferred networks specific to each of the 11 cancer types in the data using the graphical lasso (Friedman *et al.*, 2008). Without using biological prior knowledge, the networks captured many known links but also showed many differences between cancer types. We used DiffNet to assess the significance of differences between cancer types (Fig. 1B). These results account for uncertainty in network estimation and adjust for multiple comparisons due to testing across cancer-type pairs. Some pairs of cancers do not show significant network differences at the 1% FDR level, including READ/COAD, LUAD/LUSC, LUSC/HNSC and UCEC/BLCA (the first three pairs are known to be closely related). Our results show that with these exceptions, most cancer types indeed appear to be significantly different at the network level.

We also carried out a similar but ‘local’ analysis using only proteins belonging to specific pre-defined pathways (listed in Supplementary Table S2) rather than all proteins together as above. This analysis can be thought of as similar to a gene-set test, but one that captures differences in partial correlation patterns between gene set members (see Städler and Mukherjee, 2015). This broadly confirmed the global view, but revealed a number of pathway-specific insights (Supplementary Fig. S2).

### 3.2 Subtype identification using MixGlasso

**Simulation study.** We tested MixGlasso using a simulation strategy that, as above, aimed to mimic the TCGA protein data. In brief this was done by defining clusters corresponding to the cancer types in the TCGA data, with cluster-specific parameters given by estimates from the TCGA data. Data were then generated from the resulting model. This allowed us to mimic some features of the real data (including sample size and dimensionality) while allowing access to ground-truth cluster assignments. We compared MixGlasso to the following approaches:

- *K-means clustering (‘K-means’)*.
- *Hierarchical clustering* (hclust).
- *Conventional Gaussian mixture model (‘Gaussian Mixture’)*. This is a classical Gaussian mixture model with unconstrained covariance matrices, fitted using maximum likelihood.
- *Gaussian mixture model with model selection* (mclust). This is a model-based approach due to Fraley *et al.* (2012) that is one of the most widely used tools for mixture modelling.
- *Penalized Gaussian mixture model (‘Gaussian Mixture (penalised)’)*. This is a Gaussian mixture model with $\ell_{1}$-penalized inverse covariance matrices. The penalty is as described in Zhou *et al.* (2009), but without penalization of the means. The key difference to MixGlasso is that the penalty does not adapt to the cluster-specific sample size and scale.

**Fig. 2.**
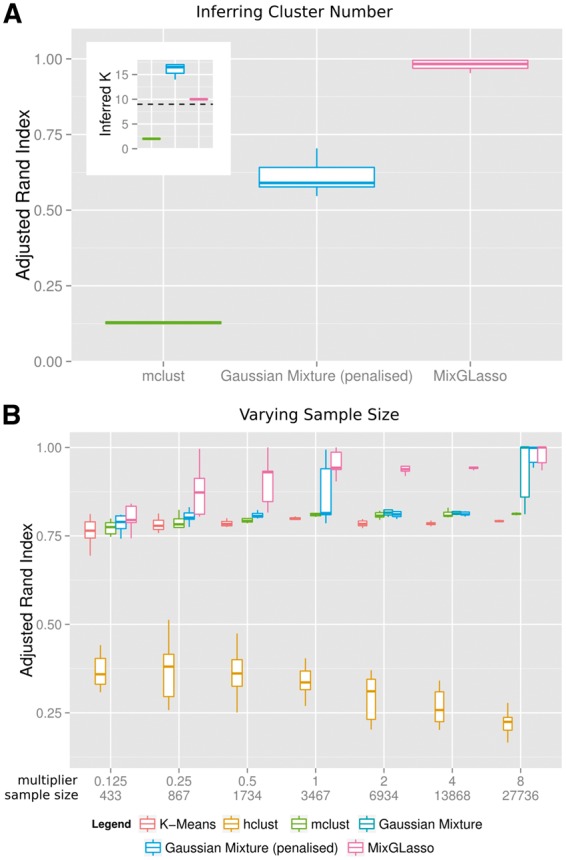
MixGlasso, simulation study. (**A**) Boxplot showing adjusted Rand index of estimated cluster assignments compared to true cluster assignments, over 10 simulated datasets, each with total sample size n=3,467. Data were generated from a Gaussian mixture model aiming to mimic characteristics of the TCGA protein data (see SI for details). Inset boxplot displays the inferred number of clusters using the Bayesian Information Criterion (BIC) and the dashed horizontal line shows the true number of clusters in the simulation (*K* = 9). (**B**) Boxplot showing adjusted Rand index (with respect to true cluster assignments) over 10 simulated datasets with varying total sample size *n* (number of clusters fixed to *K* = 9). See Main Text for a description of the different clustering methods. Note that the conventional Gaussian mixture model can only be fitted for large sample sizes (Color version of this figure is available at *Bioinformatics* online.)

We first investigated the estimation of the number *K* of clusters. There is a vast literature on this topic and a wide range of heuristics used in practice. We are interested in addressing the high-dimensional issues inherent to network-based clustering. For a focused comparison, we therefore compared MixGlasso against mclust and the penalized Gaussian mixture model as these methods make similar modeling assumptions to MixGlasso but differ in how they handle the high-dimensional aspect. This allows for a direct comparison, using the same model selection approach to select the number of clusters in each case. All the methods are likelihood-based and we used the Bayesian Information Criterion or BIC to set the number of clusters. The penalized Gaussian mixture model used the same regularization parameter $\lambda_{\text{uni}}$ as MixGlasso (see Section 2.2). Figure 2A shows the results of the analysis. MixGlasso comes closest to determining the correct number of clusters (*K *=* *9), and also agrees well with true cluster assignments.

Next, we considered accuracy of cluster assignments as a function of sample size (Fig. 2B). We included K-means and hclust in the comparison. To avoid confounding by choice of model selection heuristic, we treated the true number of clusters as known and focused on comparing cluster assignments. As expected, performance improves with sample size; however, at smaller sample sizes, MixGlasso tends to outperform the other methods, and only at large sample sizes do the classical mixture models catch up.

Several of the methods, including MixGlasso, make Gaussianity assumptions. We therefore performed additional simulations using a non-Gaussian model (Supplementary Fig. S1B, see also SI Section 4); MixGlasso appears reasonably robust to departures from Gaussianity.

**Fig. 3.**
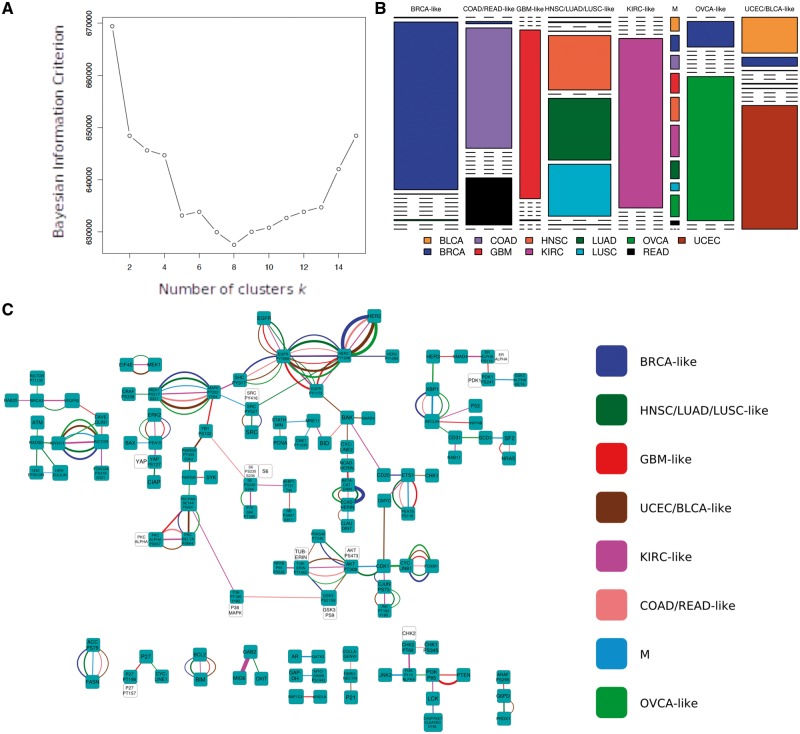
MixGlasso analysis of TCGA protein data. (**A**) Model selection using the Bayesian Information Criterion (BIC). (**B**) Mosaic plot of cluster membership in the K=8 novel clusters with respect to the 11 TCGA cancer types. Dashed lines represent absent cancer types; column width is proportional to cluster-specific sample size. (**C**) Cluster-specific networks. Strength of links is indicated by edge thickness, while color indicates cluster in which the link was observed; only relatively strong links are shown. Nodes in white are related nodes that were highly correlated and merged prior to network analysis. The adjacent correlated (green) node was then used for network generation. [Edges with partial correlations above 0.25 are shown] (Color version of this figure is available at *Bioinformatics* online.)

**TCGA data.** First we used BIC to select the number of clusters *K*; this showed an optimum at K=8 (Fig. 3A). We tested stability by iterative subsampling; at each iteration, we removed 1/4 of the data samples at random, and then scored between-iteration concordance between assignments (using the adjusted Rand index). MixGlasso is highly stable with an adjusted Rand index of 0.94 ± 0.03 over subsamples. To assess the stability of individual clusters we additionally used a resampling approach due to Hennig (2007) that quantifies individual cluster quality (Supplementary Fig. S3A).

Most of the clusters show a dominant cancer or lineage membership; accordingly we named the clusters after the dominant cancer(s). An exception is a cluster that spans 10/11 cancers and has no dominant cancer; we named this cluster ‘M’ (for mixed) and discuss it below. Figure 3C shows networks across all clusters, indicating cluster-specific as well as shared edges. Note that the partial correlations shown here are obtained by first estimating the cluster membership using MixGlasso, then merging proteins whose expression profiles are highly correlated with those of their phosphorylated forms, and finally re-estimating the networks using the graphical lasso. The merging step is necessary as otherwise the network would be dominated by (trivial) partial correlations between proteins and their phosphorylated forms. We also created a mosaic plot comparing the proposed clustering with the known cancer types (Fig. 3B, see also Supplementary Fig. S3B for the corresponding cross-tabulation of the sample numbers). Comparison between cluster assignments and known cancer types shows an adjusted Rand index of 0.73.

We further compared our approach to results reported in Hoadley *et al.* (2014) obtained using a consensus clustering approach (COCA) applied to six different TCGA data types (whole-exome DNA sequencing, DNA copy-number variation, DNA methylation, genome-wide mRNA gene expression, and RPPA protein expression for 131 proteins). Supplementary Fig. S5 shows a comparison of the MixGlasso clustering with COCA, as well as with the Pearson-Ward clustering forming part of the input to COCA (PW-131), and the clustering in Akbani *et al.* (2014) (PW-181). The comparison focuses on 2,809 TCGA samples in common across the analyses and a number of differences are apparent. Interestingly, of the three clusterings that use protein data only, MixGlasso most closely agrees with the integrative consensus clustering (adjusted Rand Index 0.76, vs 0.65 for PW-131 and 0.6 for PW-181).

**Insights from MixGlasso.** The results below serve to illustrate the nature of the output that can be immediately obtained from MixGlasso. We note that further work will be needed to better understand these results and to more comprehensively test the robustness of the reported patterns.

*A pan-cancer, protein-defined cluster.*

Cluster M spans 10/11 of the TCGA cancer types and there is no clearly dominant cancer type. Cluster M has a distinct protein abundance profile (Supplementary Fig. S4), with (among other proteins) high abundance of Yap and Taz (which share the same target and overlapping functions) and low levels of ATM, RBM15 and MTOR, as well as low levels of some phospho-proteins (with the exception of pp27 and pCHK2) which could be a signal of DNA damage and cell cycle arrest.

*A subset of ‘super basal’ breast cancers.*

**Fig. 4.**
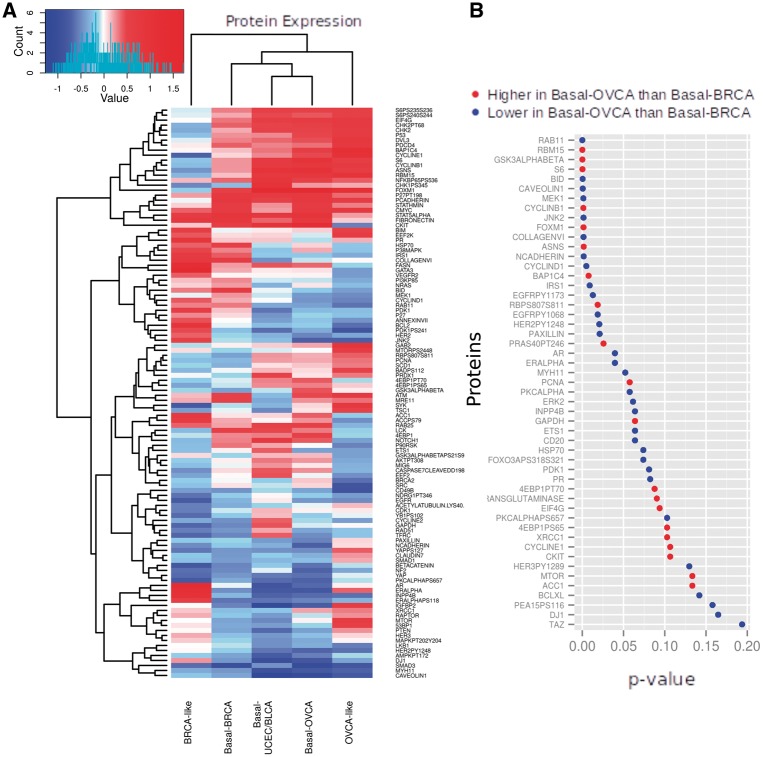
OVCA-like basal samples. A subset of basal breast samples cluster with ovarian samples. (**A**) Mean normalized protein levels for basal breast samples that are clustered with OVCA samples (Basal-OVCA), with BRCA samples (Basal-BRCA), with UCEC/BLCA (Basal-UCEC/BLCA), as well as the BRCA-like and OVCA-like clusters. (**B**) Comparison of normalized protein levels between Basal-OVCA (67 out of total 120 basal samples) and Basal-BRCA using a paired t-test. Only proteins with *P*-value < 0.2 are displayed. [Data were globally standardized prior to testing such that each protein had zero mean and unit variance over all samples] (Color version of this figure is available at *Bioinformatics* online.)

From *n* = 747 BRCA samples in our study, 70 are assigned to the cluster that contains the majority of the OVCA samples (‘OVCA-like’), rather than to the cluster that contains the majority of BRCA samples (‘BRCA-like’). Most of these samples (67/70) belong to the basal subtype of breast cancer. Genomic similarities between basal and high-grade serous ovarian samples have been previously noted (The Cancer Genome Atlas Network, 2012). However, we find that only a subset of basal breast samples (67 out of a total of 120 basal samples in the study) appear in the OVCA-like cluster. We compared these samples (‘basal-OVCA’) to the basal samples that remain clustered with BRCA samples (‘basal-BRCA’; Fig. 4A, B). We find that in basal-OVCA, a number of proteins show significantly different abundance patterns that are typically associated with the basal subtype; this includes lower abundance of the three markers for ‘triple-negative’ breast cancer (ER*α*, PR and pHER2; total HER2 is lower among basal-OVCA, but not significantly so), as well as higher abundance of several cyclins, including CyclinE1 and CyclinB1, and higher activation of several PI3K pathway proteins, including total S6 and two forms of p4EBP1 (Fig. 4B) as well as FoxM1. In each of these cases, the characteristics that define the basal breast cancer subtype seem to be amplified in the OVCA-basal samples.

## 4 Discussion and conclusions

Large datasets are often heterogeneous and often such data are in a sense collections of smaller datasets with non-identical underlying models. Furthermore, models capable of capturing even a very approximate view of molecular interplay tend to be relatively complex. We think that these two factors mean that high-dimensional statistical ideas will play a central role in the emerging areas of precision, stratified and systems medicine, even when sample sizes become much larger than is currently the case. The methods we described should be applicable to most continuous molecular data types and can be run directly with minimal tuning.

We did not consider the case of truly large *p* in this paper. The methods and ideas we discussed should be relevant to future work aimed at that setting. At the same time, the challenges involved should not be underestimated. As we showed, analyses involving moderate numbers of variables but capturing patterns of interplay are already challenging and require care.

Heterogeneity at one level of biology does not necessarily imply heterogeneity at another. For example, differential expression may or may not be accompanied by differences in patterns of interplay, and differences in gene expression may or may not be accompanied by changes in functional protein levels. Thus, although the intertumoral genomic heterogeneity of cancers is now well established, the question of whether such heterogeneity appears also at the level of signaling proteins has remained open. DiffNet applied to high-quality protein data over thousands of patient samples supported the notion that cancers differ at the protein network level. Furthermore, we showed how MixGlasso could be used to reveal new examples of commonalities across subtypes, where a subset of samples from a certain type is closer to samples from a second type than it is to other samples of the first type.

It is important to note that the graphical models in our analyses are *not* causal models *per se* and links may be driven by non-causal associations, e.g. co-regulation by an unobserved confounder (despite this caveat, many high-scoring links in the networks appeared consistent with the biochemical literature). However, our approaches are rooted in a principled, likelihood-based framework and it should be possible to extend the methods towards causal models and interventional data.

## Funding

This work was supported in part by the National Institutes of Health (NCI P30 CA016672, U54HG008100, UO1CA168394 and U24CA199461 to G.B.M.) and the UK Medical Research Council (MC_UP_1302/1 and MC_UP_1302/3).

*Conflict of Interest*: none declared.

## Supplementary Material

Supplementary Data

Supplementary Figures

## References

[btx322-B1] Akbani R. et al (2014) A pan-cancer proteomic perspective on The Cancer Genome Atlas. Nat. Commun., 5, 3887. 24871328 10.1038/ncomms4887 PMC4109726

[btx322-B2] Andersson R. et al (2014) An atlas of active enhancers across human cell types and tissues. Nature, 507, 455–461. 24670763 10.1038/nature12787 PMC5215096

[btx322-B3] Chen S.X., Qin Y.-L. (2010) A two-sample test for high-dimensional data with applications to gene-set testing. Ann. Stat., 38, 808–835.

[btx322-B4] De Smet R., Marchal K. (2010) Advantages and limitations of current network inference methods. Nat. Rev. Microbiol., 8, 717–729. 20805835 10.1038/nrmicro2419

[btx322-B5] Fraley C. et al (2012) mclust version 4 for R: Normal mixture modeling for model-based clustering, classification, and density estimation. Technical Report Technical Report No. 597, Department of Statistics, University of Washington.

[btx322-B6] Friedman J. et al (2008) Sparse inverse covariance estimation with the graphical lasso. Biostatistics, 9, 432–441. 18079126 10.1093/biostatistics/kxm045 PMC3019769

[btx322-B7] Hennig C. (2007) Cluster-wise assessment of cluster stability. Comput. Stat. Data Anal., 52, 258–271.

[btx322-B8] Hoadley K.A. et al (2014) Multiplatform analysis of 12 cancer types reveals molecular classification within and across tissues of origin. Cell, 158, 929–944. 25109877 10.1016/j.cell.2014.06.049 PMC4152462

[btx322-B9] Martens J.H., Stunnenberg H.G. (2013) BLUEPRINT: mapping human blood cell epigenomes. Haematologica, 98, 1487–1489. 24091925 10.3324/haematol.2013.094243 PMC3789449

[btx322-B10] Mukherjee S., Hill S.M. (2011) Network clustering: probing biological heterogeneity by sparse graphical models. Bioinformatics, 27, 994–1000. 21317141 10.1093/bioinformatics/btr070 PMC3065697

[btx322-B11] Pan W., Shen X. (2007) Penalized model-based clustering with application to variable selection. J. Mach. Learn. Res., 8, 1145–1164.

[btx322-B12] Schäfer J., Strimmer K. (2005) A shrinkage approach to large-scale covariance matrix estimation and implications for functional genomics. Stat. Appl. Genet. Mol. Biol., 4. 10.2202/1544-6115.1175 16646851

[btx322-B13] Shen R. et al (2009) Integrative clustering of multiple genomic data types using a joint latent variable model with application to breast and lung cancer subtype analysis. Bioinformatics, 25, 2906–2912. 19759197 10.1093/bioinformatics/btp543 PMC2800366

[btx322-B14] Städler N., Mukherjee S. (2013) Penalized estimation in high-dimensional hidden Markov models with state-specific graphical models. Ann. Appl. Stat., 7, 2157–2179.

[btx322-B15] Städler N., Mukherjee S. (2015) Multivariate gene-set testing based on graphical models. Biostatistics, 16, 47–59. 24974316 10.1093/biostatistics/kxu027

[btx322-B16] Städler N., Mukherjee S. (2017) Two-sample testing in high-dimensional models. J. R. Stat. Soc. Ser. B, 79, 225–246.

[btx322-B17] The Cancer Genome Atlas. (2012) Comprehensive molecular portraits of human breast tumours. Nature, 490, 61–70. 23000897 10.1038/nature11412 PMC3465532

[btx322-B18] Zhou H. et al (2009) Penalized model-based clustering with unconstrained covariance matrices. Electronic J. Stat., 3, 1473. 10.1214/09-EJS487 PMC2867492 20463857

