# Supplementary material for: Molecular heterogeneity at the network level: high-dimensional testing, clustering and a TCGA case study
Source: Bioinformatics. 2017 May 23;33(18):2890–6. doi: 10.1093/bioinformatics/btx322 (PMC5590725; doi:10.1093/bioinformatics/btx322)
Supplement: Supplementary Data [file btx322_suppl_stadler_et_al_si.pdf]

Molecular heterogeneity at the network level: high-dimensional testing, clustering and a TCGA case study

Supplementary information and tables

1 EM algorithm for MixGlasso optimization

We outline the EM algorithm used for optimization of the MixGlasso problem (see Main Text). We set the component responsibilities as

$$u_k(i) = \mathrm{P}[S_i = k | X_i, \Theta_K], \quad i = 1, \ldots, n \text{ and } k = 1, \ldots, K,$$

and introduce the sufficient statistics

$$\mathbf{T}_1^{u_k} = \sum_{i=1}^{n} u_k(i) X_i, \quad \mathbf{T}_2^{u_k} = \sum_{i=1}^{n} u_k(i) X_i X_i^T.$$

The EM algorithm comprises the following steps:

1. Initialization: Perform k-means clustering (100 random starts; maximal number of iterations set to 1000) to obtain initial cluster assignments and initial estimate $\hat{\Theta}_{K,\lambda_{\mathrm{uni}}}$.
2. Expectation step: Based on the current estimate $\hat{\Theta}_{K,\lambda_{\mathrm{uni}}}$, update the component responsibilities:

   $$\hat{u}_k(i) = \mathrm{P}[S_i = k | X_i, \hat{\Theta}_{K,\lambda_{\mathrm{uni}}}], \quad i = 1, \ldots, n \text{ and } k = 1, \ldots, K.$$

3. Maximization step: Update parameter $\hat{\Theta}_{K,\lambda_{\mathrm{uni}}}$:
   - Component probabilities: $\hat{\pi}_k = \hat{n}_k / n$, where $\hat{n}_k = \sum_{i=1}^{n} \hat{u}_k(i)$ is the estimated sample size for component k.
   - Component-specific means: $\hat{\mu}_k = \mathbf{T}_1^{\hat{u}_k} / \hat{n}_k$.

- Component-specific concentration matrices:

$$\hat{\Omega}_k = \underset{\Omega_k}{\operatorname{argmin}} -\log|\Omega_{\rm k}| + {\rm tr}(\Omega_{\rm k}\mathbf{S}^{\hat{\rm u}_{\rm k}}) + 2\frac{\lambda_{\rm uni}}{\hat{\rm n}_{\rm k}}\hat{\pi}_{\rm k}^{1/2}\sum_{\rm j\neq j'}|\Omega_{\rm k;jj'}|/\sqrt{\Omega_{\rm k;jj}\Omega_{\rm k;j'j'}}, \qquad (1.1)$$

with $\mathbf{S}^{\hat{u}_k} = \frac{1}{\hat{n}_k}\mathbf{T}_2^{\hat{u}_k} - \hat{\mu}_k\hat{\mu}_k^T$.

4. Repeat steps 1 and 2 until (i) relative change in $\hat{\Sigma}_k$'s is smaller than 10^{-3} or (ii) any cluster fell below a minimum size, i.e. $\min_k \hat{n}_k < 5$.

Computation of (1.1) is non-standard and can be solved using the "scaled graphical Lasso" presented in Städler and Mukherjee (2013). The scaled graphical Lasso objective is

$$-\log|\Omega| + {\rm tr}(\mathbf{S}\Omega) + \rho\sum_{\rm j\neq j'}|\Omega_{\rm jj'}|/\sqrt{\Omega_{\rm jj}\Omega_{\rm j'j'}}.$$

Optimization can be performed by setting $\Omega^{(0)} = {\rm Diag}(\mathbf{S})^{-1}$ and iteratively calling the standard graphical Lasso algorithm according to

$$\Omega^{(i+1)} = \underset{\Omega}{\operatorname{argmin}} -\log|\Omega| + {\rm tr}(\mathbf{S}\Omega) + \sum_{\rm j\neq j'}\frac{\rho}{\sqrt{\Omega^{(\rm i)}_{\rm jj}\Omega^{(\rm i)}_{\rm j'j'}}}|\Omega_{\rm jj'}| \quad ({\rm i}=0,1,2,\ldots).$$

Note, that the **R**-package `glasso` allows specification of different penalty levels for different entries in Ω (here: $\rho/\sqrt{\Omega^{(i)}_{jj}\Omega^{(i)}_{j'j'}}$, $j, j' \in \{1,\ldots,p\}$, $j \neq j'$).

2 Simulation Setup

2.1 Simulation model for testing the MixGlasso

In order to test the effectiveness of the MixGlasso method presented in this paper, we devised a network-based simulation model that borrows from the characteristics of the TCGA data to present a reasonable facsimile of real data. We proceeded by first applying the Pearson-Ward clustering of the TCGA data described in Akbani *et al.* (2014). For each of the $K = 9$ clusters, we record the mean μ_k and the variance σ_k^2 over all proteins. Let $S_i \in \{1,\ldots,K\}$ be latent discrete labels indicating cluster membership of the ith observation, i.e. $S_i = k$ if observation i belongs to component (or cluster) k. The component probabilities are $\pi_k = {\rm P}(S_i = k)$. We then sample from the mixture model:

$$X_i \mid S_i = k \sim \mathcal{N}(\mu_k, \Sigma_k) \qquad (2.2)$$

where $X_i \in \mathbb{R}^p$ is the ith simulated vector. The component probabilities π_k are based on the frequencies of the clusters in the TCGA data. Note that each Σ_k is a full covariance matrix generated with a given sparsity s determining the number of off-diagonal zero entries, and the off-diagonal non-zero entries are drawn from a beta distribution with parameters $a = b = 5$. The covariance matrix is then adjusted so that the variances correspond to the σ_k^2 vectors obtained from the TCGA data. This procedure serves two purposes: 1) using the σ_k^2 from the TCGA data allows us to explore the situation where the variances are at different scales. 2) Generating the off-diagonal covariances in this way allows us to control sparsity as one of the parameters of the model. In the results presented in this paper, we have set $s = 0.05$, meaning that only 5% of the off-diagonal entries are non-zero. More dense covariance matrices did decrease the performance of MixGlasso slightly in our preliminary studies; however, we note that that the performance of other methods also decreased, and thus relative performance was unaffected.

2.2 Simulation model for testing DiffNet

The procedure for generating the simulation data for evaluating our DiffNet method for high-dimensional network-based statistical hypothesis testing is based on the TCGA dataset described in Akbani *et al.* (2014). The following procedure generates two datasets that can then be compared using DiffNet:

1. Consider LUAD and GBM samples in the TCGA dataset. The sample sizes are $n_{\text{LUAD}} = 237$, $n_{\text{GBM}} = 215$ and the dimensionality is $p = 181$.

2. Compute inverse covariance matrices separately from LUAD and GBM samples by applying the graphical Lasso on centered and scaled data. The penalty λ is chosen using 10-fold cross-validation, and the smallest coefficients are thresholded such that there are not more than p non-zero elements. We retrieve the correlation matrices Σ_{LUAD} and Σ_{GBM}.

3. Under the null-hypothesis: generate $n_1 \times p$ data-matrix $\mathbf{X}_1$ according to $\mathcal{N}(0, \Sigma_{\text{LUAD}})$ and $n_2 \times p$ data-matrix $\mathbf{X}_2$ according to $\mathcal{N}(0, \Sigma_{\text{LUAD}})$. Here, the sample sizes are multiples of n_{LUAD} and n_{GBM}, i.e. $n_1 = \lceil m \times n_{\text{LUAD}} \rceil$ and $n_2 = \lceil m \times n_{\text{GBM}} \rceil$ where $m \in \{0.2, 0.3, \ldots, 1.5\}$ is the sample size multiplier.

4. Under the alternative hypothesis (rejection of the null hypothesis): generate $n_1 \times p$ data-matrix $\mathbf{X}_1$ according to $\mathcal{N}(0, \Sigma_{\text{LUAD}})$ and $n_2 \times p$ data-matrix $\mathbf{X}_2$ according to $\mathcal{N}(0, \Sigma_{\text{GBM}})$, where $n_1 = \lceil m \times n_{\text{LUAD}} \rceil$ and $n_2 = \lceil m \times n_{\text{GBM}} \rceil$ ($m \in \{0.2, 0.3, \ldots, 1.5\}$).

   We then compare the datasets $\mathbf{X}_1$ and $\mathbf{X}_2$ (under null- and alternative-hypothesis) using the following two-sample tests:

- LRT(Asym): conventional likelihood-ratio test (p-values based on asymptotic $\chi^2_{p(p+1)/2}$-distribution)
- LRT(Perm): permutation-based likelihood-ratio test (p-values based on 200 random permutations of condition labels)
- Mult.FisherZ: use Fisher's Z-transform to perform two-sample comparisons for all partial correlation coefficients:

  $$\mathbf{H}_0 : \rho_{1,jj'} = \rho_{2,jj'}, \quad \text{for all } 1 \le j < j' \le p.$$

  Here, $\rho_{1,jj'}$ and $\rho_{2,jj'}$ denote condition specific partial correlation coefficients and denote by

  $$P^{\text{FisherZ}}_{jj'}, 1 \le j < j' \le p,$$

  the corresponding p-values. In order to test the "global" hypothesis, $\mathbf{H}_0 : \Omega_1 = \Omega_2$, we report

  $$P^{\text{Mult.FisherZ}} = \frac{(p-1)p}{2} \min_{j<j'}\{P^{\text{FisherZ}}_{jj'}\},$$

  which corresponds to the minimum of the Bonferroni-corrected individual p-values.
- DiffNet(SS) and DiffNet(MS): differential network with $B = 1$, respectively $B = 50$ data-splits.

The data is generated 100 times for each value $m \in \{0.2, 0.3, \ldots, 1.5\}$. For each generated dataset we report the false positive rate (FPR) and the true positive rate (TPR). Figure 1 in the Main Text shows FPR and TPR (type-I error and power) where the threshold for significance has been set at 1%, as well as AUC (area under the curve) for different values of m.

For Figure S1 (DiffNet with t_{df}-distribution), we proceed as above, but instead of sampling from a multivariate normal distribution, data are sampled from the multivariate t-distribution with degrees of freedom df $= \{\infty, 20, 10, 6, 4\}$. The sample size multiplier is fixed at $m = 1$ and performance (type-I error, power and AUC) is plotted as a function of df.

3 Differential expression and wiring analysis

Consider pathways $A_s \subset \{1, \ldots, p\}$, $s = 1, \ldots, S$, and let $\tau_{s,j}$ be the sign of protein j in pathway s, indicating whether protein j is a positive or negative regulatory component in the pathway, as defined in Akbani *et al.* (2014). For each disease type

k, $k = 1, \ldots, K$, we calculate the differential expression score and the differential wiring score as follows:

Differential Expression: Let $\hat{\mu}_j^{(k)} = \bar{\mathbf{X}}_{k,j}$ be the mean expression for protein j in disease type k. We then define the *differential expression* score for pathway s and disease type k as:

$$\mathrm{DE}_s^{(k)} = \mathrm{median}\left\{\frac{1}{|A_s|}\sum_{j\in A_s}\tau_{s,j}(\hat{\mu}_j^{(k)} - \hat{\mu}_j^{(k')}); k' \in \{1,\ldots,K\}, k' \neq k\right\}. \quad (3.3)$$

Differential Wiring: For $s = 1, \ldots, S$, $k, k' = 1, \ldots, K$, denote by $P_s^{(k,k')}$ the differential network p-value (Bonferroni corrected) comparing networks (based on only proteins belonging to pathway s) between disease types k and k'. Define the *differential wiring* score for pathway s and disease type k as the number of disease types k' with significant p-value $P_s^{(k,k')}$ at the $\alpha = 0.01$ level:

$$\mathrm{DW}_s^{(k)} = \sum_{k'\in\{1,\ldots,K\},k'\neq k} \mathbf{1}_{P_s^{(k,k')}<\alpha}. \quad (3.4)$$

4 Additional Simulation Results

Figure S1 presents an additional simulation study into the effect of analysing data that violates the Gaussian assumptions of DiffNet and MixGlasso. In order to achieve this, we replace the Gaussian distribution in (2.2) with a multi-variate t-distribution with the same μ_k and Σ_k, and varying degrees of freedom df. In the limit of $df \to \infty$, the Gaussian distribution is recovered. The multi-variate t-distribution presents heavier tails than a Gaussian distribution.

Figure S1A shows the results of applying DiffNet to this data; we note that DiffNet is well-behaved for $df > 4$. At low df values, the Type-I error of DiffNet increases slightly, but we note that it still has higher power and better ROC performance than Fisher's Z transform and the conventional (asymptotic) likelihood test.

In Figure S1B, we have tested MixGlasso on the same data. We compare performance of 3 methods: mclust, a Gaussian mixture model with ℓ_1-penalized inverse covariance matrices, and MixGlasso. For details on these methods, see the Main Text. The leftmost plot shows that MixGlasso tends to outperform the other methods in terms of adjusted Rand index with the true cluster assignments, for $df \geq 4$. Note that we have allowed the true number of cluster K to be inferred from the data along with the cluster assignments. The middle plot shows the inferred K, showing that MixGlasso is most accurate for $df \geq 4$. Interestingly, the naive penalised mixture model tends to over-estimate the number of clusters even when the Gaussian assumption is not violated, indicating that the scale-invariant nature of MixGlasso aids in determining the right number of clusters. Finally, the last plot shows the

stability of the clustering, obtained by repeated subsampling of the datasets and calculating the adjusted Rand index between the obtained cluster assignments. This shows that higher degrees of freedom generally lead to more stable clusterings.

5 Reverse phase protein array data

Proteomic data were generated by Reverse phase protein arrays (RPPA) across a total of $3,467$ patient samples including breast (BRCA, $n = 747$), colon adenocarcinoma (COAD; $n = 334$), rectal adenocarcinoma (READ; $n = 130$), renal clear cell carcinoma (KIRC, $n = 454$), high-grade serous ovarian cystadenocarcinoma (OVCA; $n = 412$), uterine corpus endometrial carcinoma (UCEC; $n = 404$), lung adenocarcinoma (LUAD, $n = 237$), head and neck squamous cell carcinoma (HNSC; $n = 212$), lung squamous cell carcinoma (LUSC; $n = 195$), bladder urothelial carcinoma (BLCA, $n = 127$) and glioblastoma multiforme (GBM, $n = 215$).

In total $p = 181$ high-quality antibodies targeting total (n=128), cleaved (n=1), acetylated (n=1) and phosphoproteins (n=51) were used (Table S1). Antibodies for phospho-HER2 and phospho-EGFR have been noticed to cross-react in RPPA, especially when the opposite molecule is present at very high levels. This mainly concerns EGFRpY1068 (but not EGFRpY1173), which cross-reacts with overexpressed HER2pY1248. Taking into account their favorable signal:noise ratio (10:1), useful information is contributed by both if expressed differentially, and they are thus both included. The antibodies encompass major functional and signaling pathways of relevance to human cancer. Pathways included are proliferation, DNA damage, EMT, hormone signaling, apoptosis, immunological, stromal, TGFα/β, transmembrane receptors, metabolism, AMPK, TSC/mTOR, PI3K/Akt, Ras/MAPK, Hippo, and Wnt/beta-catenin.

The $n = 3,467$ samples were run in 6 batches in total, resulting in potential batch effects upon merging the sets. A new algorithm, replicates-based normalization (RBN; Akbani *et al.* (2014)), was used to adjust for batch effects using control samples that were run across multiple batches; the number of control samples within each batch varied between 71 and 207. In brief, this was done by scaling and shifting data in each batch such that all control samples had zero mean and unit variance in each batch; for full details and discussion see Akbani *et al.* (2014).

Protein Name	Gene Name	Antibody Status	Antibody Origin	Antibody Source	Catalog Number	Website
14-3-3_epsilon	YWHAE	Use with Caution	Mouse	Santa Cruz	sc-23957	http://www.scbt.com/datasheet-23957-14-3-3-epsilon-8c3-antibody.html
4E-BP1_pS65	EIF4EBP1	Validated	Rabbit	CST	9456	http://www.cellsignal.com/products/9456.html
4E-BP1_pT37	EIF4EBP1	Validated	Rabbit	CST	9459	http://www.cellsignal.com/products/9459.html
4E-BP1	EIF4EBP1	Validated	Rabbit	CST	9452	http://www.cellsignal.com/products/9452.html
53BP1	TP53BP1	Under Evaluation	Rabbit	CST	4937	http://www.cellsignal.com/products/4937.html
ACC_pS79	ACACA ACACB	Validated	Rabbit	CST	3661	http://www.cellsignal.com/products/3661.html
ACC1	ACACA	Under Evaluation	Rabbit	Epitomics	1768-1	http://www.epitomics.com/products/product_info/419/Acetyl-antibody-1768-1.html
Akt_pS473	AKT1 AKT2 AKT3	Validated	Rabbit	CST	9271	http://www.cellsignal.com/products/9271.html
Akt_pT308	AKT1 AKT2 AKT3	Validated	Rabbit	CST	9275	http://www.cellsignal.com/products/9275.html
Akt	AKT1 AKT2 AKT3	Validated	Rabbit	CST	9272	http://www.cellsignal.com/products/9272.html
AMPK_alpha	PRKAA1	Use with Caution	Rabbit	CST	2532	http://www.cellsignal.com/products/2532.html
AMPK_pT172	PRKAA1	Validated	Rabbit	CST	2535	http://www.cellsignal.com/products/2535.html
AR	AR	Validated	Rabbit	Epitomics	1852-1	http://www.epitomics.com/products/product_info/259/Androgen-antibody-1852-1.html
ATM	ATM	Under Evaluation	Rabbit	Abcam	ab32420	http://www.abcam.com/atm-antibody-y170-ab32420.html
Bak	BAK1	Under Evaluation	Rabbit	Epitomics	1542-1	http://www.epitomics.com/products/product_info/83/Bak-antibody-1542-1.html
Bax	BAX	Validated	Rabbit	CST	2772	http://www.cellsignal.com/products/2772.html
Bcl-2	BCL2	Validated	Mouse	Dako	Dako M0887	http://www.dako.com/us/ar38/p102230/prod_products.htm
Bcl-xL	BCL2L1	Validated	Rabbit	CST	2762	http://www.cellsignal.com/products/2762.html
Beclin	BECN1	Use with Caution	Goat	Santa Cruz	sc-10086	http://www.scbt.com/datasheet-10086-becn1-d-18-antibody.html

beta-Catenin	CTNNB1	Validated	Rabbit	CST	9562	http://www.cellsignal.com/products/9562.html
Bid	BID	Use with Caution	Rabbit	Epitomics	1008-1	http://www.epitomics.com/products/product_info/93/Bid-antibody-1008-1.html
Bim	BCL2L11	Validated	Rabbit	Epitomics	1036-1	http://www.epitomics.com/products/product_info/94/Bim-antibody-1036-1.html
c-Jun_pS73	JUN	Validated	Rabbit	CST	9164	http://www.cellsignal.com/products/9164.html
c-Kit	KIT	Validated	Rabbit	Epitomics	1522	http://www.epitomics.com/products/product_info/158/c-Kit-CD117-antibody-1522-1.html
c-Met_pY1235	MET	Validated	Rabbit	CST	3129	http://www.cellsignal.com/products/3129.html
c-Myc	MYC	Use with Caution	Rabbit	CST	9402	http://www.cellsignal.com/products/9402.html
C-Raf_pS338	RAF1	Under Evaluation	Rabbit	CST	9427	http://www.cellsignal.com/products/9427.html
C-Raf	RAF1	Validated	Rabbit	Millipore	05-739	https://www.millipore.com/coa.nsf/a73664f9f981af8c852569b9005b4eee/5ccbe14c984f4587852573060052d7aa/$FILE/05-739-28714.pdf
Caspase-7_cleavedD198	CASP7	Use with Caution	Rabbit	CST	9491	http://www.cellsignal.com/products/9491.html
Caveolin-1	CAV1	Validated	Rabbit	CST	3238	http://www.cellsignal.com/products/3238.html
CD31	PECAM1	Validated	Mouse	Dako	M0823	http://www.dako.com/us/ar38/p102870/prod_products.htm
CD49b	ITGA2	Validated	Mouse	BD	611016	http://www.bdbiosciences.com/ pt-Product.jsp?prodId=108074&key=611016&param=search&mterms=true&from=dTable
CDK1	CDC2	Validated	Rabbit	CST	9112	http://www.cellsignal.com/products/9112.html
Chk1_pS345	CHEK1	Use with Caution	Rabbit	CST	2348	http://www.cellsignal.com/products/2348.html
Chk1	CHEK1	Under Evaluation	Rabbit	CST	2345	Discontinued, replaced by http://www.cellsignal.com/products/2360.html
Chk2_pT68	CHEK2	Under Evaluation	Rabbit	CST	2197	http://www.cellsignal.com/products/2197.html
Chk2	CHEK2	Under Evaluation	Mouse	CST	3440	http://www.cellsignal.com/products/3440.html
cIAP	BIRC2	Use with Caution	Rabbit	Millipore	07-759	https://www.millipore.com/catalogue/item/07-759
Claudin-7	CLDN7	Validated	Rabbit	Novus	NB100-91714	http://www.novusbio.com/Claudin-7-Antibody_NB100-91714.html

Collagen_VI	COL6A1	Validated	Rabbit	Santa Cruz	SC-20649	http://www.scbt.com/datasheet-20649-col6a1-h-200-antibody.html
Cyclin_B1	CCNB1	Validated	Rabbit	Epitomics	1495-1	http://www.epitomics.com/products/product_info/170/Cyclin-antibody-1495-1.html
Cyclin_D1	CCND1	Validated	Rabbit	Santa Cruz	SC-718	http://www.scbt.com/datasheet-718-cyclin-d1-m-20-antibody.html
Cyclin_E1	CCNE1	Validated	Mouse	Santa Cruz	SC-247	http://www.scbt.com/datasheet-247-cyclin-e-he12-antibody.html
DJ-1	PARK7	Validated	Rabbit	Abcam	ab76008	http://www.abcam.com/park7-dj1-antibody-ep2815y-ab76008.html
Dvl3	DVL3	Validated	Rabbit	CST	3218	http://www.cellsignal.com/products/3218.html
E-Cadherin	CDH1	Validated	Rabbit	CST	4065	Discontinued, replaced by http://www.cellsignal.com/products/3195.html
eEF2	EEF2	Use with Caution	Rabbit	CST	2332	http://www.cellsignal.com/products/2332.html
eEF2K	EEF2K	Validated	Rabbit	CST	3692	http://www.cellsignal.com/products/3692.html
EGFR_pY1068	EGFR	Use with Caution	Rabbit	CST	2234	http://www.cellsignal.com/products/2234.html
EGFR_pY1173	EGFR	Validated	Rabbit	Epitomics	1124	http://www.epitomics.com/products/product_info/187/EGFR-antibody-1124-1.html
eIF4E	EIF4E	Validated	Rabbit	CST	9742	http://www.cellsignal.com/products/9742.html
ER-alpha_pS118	ESR1	Validated	Rabbit	Epitomics	1091-1	http://www.epitomics.com/products/product_info/201/ER-antibody-1091-1.html
ER-alpha	ESR1	Validated	Rabbit	Lab Vision	RM-9101-S	https://thermo.dirxion.com/immunohistochemistry/WebProject.asp?CodeId=7.6.3.2&BookCode=ihc12flx#
ERK2	MAPK1	Under Evaluation	Rabbit	Santa Cruz	sc-154	http://www.scbt.com/datasheet-154-erk-2-c-14-antibody.html
Fibronectin	FN1	Use with Caution	Rabbit	Epitomics	1574-1	http://www.epitomics.com/products/product_info/215/Fibronectin-antibody-1574-1.html
FOXO3a	FOXO3	Use with Caution	Rabbit	CST	9467	Discontinued, replaced by http://www.cellsignal.com/products/2497.html
GAB2	GAB2	Validated	Rabbit	CST	3239	http://www.cellsignal.com/products/3239.html
GATA3	GATA3	Validated	Mouse	BD Biosciences	558686	http://www.bdbiosciences.com/ptProduct.jsp?prodId=656284&key=558686&param=search&mterms=true&from=dTable
GSK3-alpha-beta_pS21_S9	GSK3A GSK3B	Validated	Rabbit	CST	9331	http://www.cellsignal.com/products/9331.html

GSK3-alpha-beta	GSK3A GSK3B	Validated	Mouse	Santa Cruz	SC-7291	http://www.scbt.com/datasheet-7291-gsk-3alpha-beta-0011-a-antibody.html
HER2_pY1248	ERBB2	Use with Caution	Rabbit	Upstate (Millipore)	06-229	https://www.millipore.com/catalogue/item/06-229
HER2	ERBB2	Validated	Mouse	Lab Vision	MS-325-P1	https://thermo.dirxion.com/immunohistochemistry/WebProject.asp?CodeId=7.6.3.2&BookCode=ihc12flx#
HER3_pY1289	ERBB3	Use with Caution	Rabbit	CST	4791	http://www.cellsignal.com/products/4791.html
HER3	ERBB3	Validated	Mouse	Lab Vision	MS-201-P1ABX	https://thermo.dirxion.com/immunohistochemistry/WebProject.asp?CodeId=7.6.3.2&BookCode=ihc12flx#
HSP70	HSPA1A	Use with Caution	Rabbit	CST	4872	http://www.cellsignal.com/products/4872.html
IGFBP2	IGFBP2	Validated	Rabbit	CST	3922	http://www.cellsignal.com/products/3922.html
INPP4B	INPP4B	Under Evaluation	Goat	Santa Cruz	SC-12318	http://www.scbt.com/datasheet-12318-type-ii-4-phosphatase-n-20-antibody.html
IRS1	IRS1	Validated	Rabbit	Upstate (Millipore)	06-248	https://www.millipore.com/catalogue/item/06-248
JNK-pT183_pY185	MAPK8	Validated	Rabbit	CST	4668	http://www.cellsignal.com/products/4668.html
JNK2	MAPK9	Use with Caution	Rabbit	CST	4672	http://www.cellsignal.com/products/4672.html
Ku80	XRCC5	Under Evaluation	Rabbit	CST	2180	http://www.cellsignal.com/products/2180.html
LKB1	STK11	Under Evaluation	Mouse	Abcam	ab15095	http://www.abcam.com/lkb1-antibody-ley-37dg6-ab15095.html
Lck	LCK	Validated	Rabbit	CST	2752	http://www.cellsignal.com/products/2752.html
MAPK-pT202_Y204	MAPK1 MAPK3	Validated	Rabbit	CST	4377	http://www.cellsignal.com/products/4377.html
MEK1-pS217_S221	MAP2K1	Validated	Rabbit	CST	9154	http://www.cellsignal.com/products/9154.html
MEK1	MAP2K1	Validated	Rabbit	Epitomics	1235-1	http://www.epitomics.com/products/product_info/276/MEK1-antibody-1235-1.html
MIG-6	ERRFI1	Validated	Mouse	Sigma	WH0054206M1	http://www.sigmaaldrich.com/catalog/product/sigma/wh0054206m1?lang=en&region=US
Mre11	MRE11A	Use with Caution	Rabbit	CST	4847	http://www.cellsignal.com/products/4847.html
mTOR_pS2448	FRAP1	Use with Caution	Rabbit	CST	2971	http://www.cellsignal.com/products/2971.html
mTOR	FRAP1	Validated	Rabbit	CST	2983	http://www.cellsignal.com/products/2983.html

N-Cadherin	CDH2	Validated	Rabbit	CST	4061	http://www.cellsignal.com/products/4061.html
NF-kB-p65_pS536	NFKB1	Use with Caution	Rabbit	CST	3033	http://www.cellsignal.com/products/3033.html
NF2	NF2	Use with Caution	Rabbit	SDI/ Novus	2271.00.02	http://www.novusbio.com/ Merlin-Antibody_22710002.html
Notch1	NOTCH1	Validated	Rabbit	CST	3268	http://www.novusbio.com/ Merlin-Antibody_22710002.html
P-Cadherin	CDH3	Under Evaluation	Rabbit	CST	2130	http://www.cellsignal.com/products/2130.html
p27_pT157	CDKN1B	Use with Caution	Rabbit	R&D	AF1555	http://www.rndsystems.com/Products/AF1555
p27	CDKN1B	Validated	Rabbit	Epitomics	1591-1	http://www.epitomics.com/products/product_info/ 311/p27-Kip1-antibody-1591-1.html
p38_MAPK	MAPK14	Validated	Rabbit	CST	9212	http://www.cellsignal.com/products/9212.html
p38_pT180_Y182	MAPK14	Validated	Rabbit	CST	9211	http://www.cellsignal.com/products/9211.html
p53	TP53	Under Evaluation	Rabbit	CST	9282	http://www.cellsignal.com/products/9282.html
p70S6K_pT389	RPS6KB1	Validated	Rabbit	CST	9205	http://www.cellsignal.com/products/9205.html
p70S6K	RPS6KB1	Validated	Rabbit	Epitomics	1494-1	http://www.epitomics.com/products/product_info/ 321/p70-antibody-1494-1.html
p90RSK-pT359_S363	RPS6KA1	Use with Caution	Rabbit	CST	9344	http://www.cellsignal.com/products/9344.html
PAI-1	SERPINE1	Under Evaluation	Mouse	BD Bio-sciences	612024	http://www.bdbiosciences.com/ ptProduct.jsp?prodId=106729&key=612024& param=search&mterms=true&from=dTable
Paxillin	PXN	Use with Caution	Rabbit	Epitomics	1500-1	http://www.epitomics.com/products/product_info/ 334/Paxillin-antibody-1500-1.html
PCNA	PCNA	Use with Caution	Mouse	Abcam	ab29	http://www.abcam.com/ pcna-antibody-pc10-proliferation-marker-ab29.html
PDK1_pS241	PDK1	Validated	Rabbit	CST	3061	http://www.cellsignal.com/products/3061.html
Pea-15	PEA15	Validated	Rabbit	CST	2780	http://www.cellsignal.com/products/2780.html
PI3K-p110-alpha	PIK3CA	Use with Caution	Rabbit	CST	4255	http://www.cellsignal.com/products/4255.html
PKC-alpha_pS657	PRKCA	Use with Caution	Rabbit	Upstate (Milli-pore)	06-822	https://www.millipore.com/catalogue/ item/06-822

PKC-alpha	PRKCA	Validated	Mouse	Upstate (Millipore)	05-154	https://www.millipore.com/catalogue/item/05-154
PKC-delta_pS664	PRKCD	Validated	Rabbit	Millipore	07-875	https://www.millipore.com/catalogue/item/07-875
PR	PGR	Validated	Rabbit	Epitomics	1483-1	http://www.epitomics.com/products/product_info/354/Progesterone-antibody-1483-1.html
PRAS40_pT246	AKT1S1	Validated	Rabbit	Biosource	441100G	http://products.invitrogen.com/ivgn/product/441100G?ICID=search-441100g
PTEN	PTEN	Validated	Rabbit	CST	9552	http://www.cellsignal.com/products/9552.html
Rad50	RAD50	Validated	Mouse	Millipore	05-525	https://www.millipore.com/catalogue/item/05-525
Rad51	RAD51	Under Evaluation	Mouse	Chem Biotech	na 71	
Rb_pS807_S811	RB1	Validated	Rabbit	CST	9308	http://www.cellsignal.com/products/9308.html
S6_pS235_S236	RPS6	Validated	Rabbit	CST	2211	http://www.cellsignal.com/products/2211.html
S6_pS240_S244	RPS6	Validated	Rabbit	CST	2215	http://www.cellsignal.com/products/2215.html
S6	RPS6	Under Evaluation	Rabbit	CST	2217	http://www.cellsignal.com/products/2217.html
Shc_pY317	SHC1	Validated	Rabbit	CST	2431	http://www.cellsignal.com/products/2431.html
Smad1	SMAD1	Validated	Rabbit	Epitomics	1649-1	http://www.epitomics.com/products/product_info/388/Smad1-antibody-1649-1.html
Smad3	SMAD3	Validated	Rabbit	Epitomics	1735-1	http://www.epitomics.com/products/product_info/454/Smad3-antibody-1735-1.html
Smad4	SMAD4	Validated	Mouse	Santa Cruz	sc-7966	http://www.scbt.com/table-smad4.html
Src_pY416	SRC	Use with Caution	Rabbit	CST	2101	http://www.cellsignal.com/products/2101.html
Src_pY527	SRC	Validated	Rabbit	CST	2105	http://www.cellsignal.com/products/2105.html
Src	SRC	Validated	Mouse	Upstate (Millipore)	05-184	https://www.millipore.com/catalogue/item/05-184
STAT3_pY705	STAT3	Validated	Rabbit	CST	9131	http://www.cellsignal.com/products/9131.html
STAT5-alpha	STAT5A	Validated	Rabbit	Epitomics	1289-1	http://www.epitomics.com/products/product_info/398/Stat-5-antibody-1289-1.html
Stathmin	STMN1	Validated	Rabbit	Epitomics	1972-1	http://www.epitomics.com/products/product_info/701/Stathmin-antibody-1972-1.html

Syk	SYK	Validated	Mouse	Santa Cruz	sc-1240	http://www.scbt.com/ datasheet-1240-syk-4d10-antibody.html
Tuberin	TSC2	Validated	Rabbit	Epitomics	1613-1	http://www.epitomics.com/products/product_info/ 411/TSC2-Tuberin-antibody-1613-1.html
VEGFR2	KDR	Validated	Rabbit	CST	2479	http://www.cellsignal.com/products/2479.html
XRCC1	XRCC1	Under Evaluation	Rabbit	CST	2735	http://www.cellsignal.com/products/2735.html
YAP_pS127	YAP1	Under Evaluation	Rabbit	CST	4911	http://www.cellsignal.com/products/4911.html
YAP	YAP1	Under Evaluation	Rabbit	Santa Cruz	sc-15407	http://www.scbt.com/ datasheet-15407-yap-h-125-antibody.html
YB-1_pS102	YBX1	Validated	Rabbit	CST	2900	http://www.cellsignal.com/products/2900.html
YB-1	YBX1	Validated	Rabbit	SDI	1725.00.02	http://www.novusbio.com/ YB1-Antibody_17250002.html
ASNS	ASNS	Validated	Rabbit	Sigma	HPA-029318	http://www.sigmaaldrich.com/catalog/product/ sigma/hpa029318?lang=en&region=US
EGFR	EGFR	Validated	Rabbit	CST	2232	http://www.cellsignal.com/products/2232.html
4E-BP1_pT70	EIF4EBP1	Use with Caution	Rabbit	CST	9455	http://www.cellsignal.com/products/9455.html
A-Raf_pS299	ARAF	Use with Caution	Rabbit	CST	4431	http://www.cellsignal.com/products/4431.html
Annexin_VII	ANXA7	Validated	Mouse	BD Bio-sciences	610668	http://www.bdbiosciences.com/ ptProduct.jsp?prodId=25306&key=610668& param=search&mterms=true&from=dTable
ARID1A	ARID1A	Validated	Mouse	Abgent	AT1188a	Discontinued
B-Raf	BRAF	Use with Caution	Mouse	Santa Cruz	sc-5284	http://www.scbt.com/ datasheet-5284-raf-b-f-7-antibody.html
Bad_pS112	BAD	Validated	Rabbit	CST	9291	http://www.cellsignal.com/products/9291.html
Bap1 c-4	BAP1	Ender Evaluation	Mouse	Santa Cruz	sc-28383	http://www.scbt.com/ datasheet-28383-bap1-c-4-antibody.html
BRCA2	BRCA2	Use with Caution	Rabbit	CST	9012	http://www.cellsignal.com/products/9012.html
CD20	MS4A1	Use with Caution	Rabbit	Epitomics	1632	http://www.epitomics.com/products/product_info/ 135/CD20-antibody-1632-1.html
Cyclin_E2	CCNE2	Use with Caution	Rabbit	Epitomics	1142	http://www.epitomics.com/products/product_info/ 173/Cyclin-antibody-1142-1.html
eIF4G	EIF4G1	Use with Caution	Rabbit	CST	2498	http://www.cellsignal.com/products/2498.html
FASN	FASN	Validated	Rabbit	CST	3180	http://www.cellsignal.com/products/3180.html
FOXO3a-pS318_S321	FOXO3	Use with Caution	Rabbit	CST	9465	http://www.cellsignal.com/products/3180.html

FoxM1	FOXM1	Validated	Rabbit	CST	5436	http://www.cellsignal.com/products/5436.html
G6PD	G6PD	Validated	Mouse	Santa Cruz	sc-373887	http://www.scbt.com/ datasheet-373887-g6pd-g-6-antibody.html
GAPDH	GAPDH	Use with Caution	Mouse	Ambion	AM4300	http://products.invitrogen.com/ivgn/product/ AM4300?ICID=search-am4300
GSK3_pS9	GSK3A GSK3B	Validated	Rabbit	CST	9336	http://www.cellsignal.com/products/9336.html
Heregulin-	NRG1	Validated	Rabbit	CST	2573	http://www.cellsignal.com/products/2573.html
MYH11	MYH11	Validated	Rabbit	SDI/ Novus	21370002	http://www.novusbio.com/ Myosin-heavy-chain-11-Antibody_21370002.html
Myosin IIa pS1943	MYH9	Validated	Rabbit	CST	5026	http://www.cellsignal.com/products/5026.html
N-Ras	NRAS	Validated	Mouse	Santa Cruz	sc-31	http://www.scbt.com/ datasheet-31-n-ras-f155-antibody.html
NDRG1_pT346-	NDRG1	Validated	Rabbit	CST	3217	http://www.cellsignal.com/products/3217.html
p21	CDKN1A	Validated	Rabbit	Santa Cruz	SC-397	http://www.scbt.com/ datasheet-397-p21-c-19-antibody.html
p27_pT198	CDKN1B	Validated	Rabbit	Abcam	ab64949	http://www.abcam.com/ p27-kip-1-phospho-t198-antibody-ab64949.html
p90RSK	RPS6KA1	Use with Caution	Rabbit	CST	9347	http://www.cellsignal.com/products/9347.html
PDCD4	PDCD4	Use with Caution	Rabbit	Rockland	600-401-965	http://www.rockland-inc.com/store/ Apoptosis-Antibodies-600-401-965-O4L_18137.aspx
PDK1	PDK1	Validated	Rabbit	CST	3062	http://www.cellsignal.com/products/3062.html
PEA15_pS116	PEA15	Validated	Rabbit	Invitrogen	44-836G	http://products.invitrogen.com/ivgn/product/ 44836G?ICID=search-44836g
PI3K-p85	PIK3R1/2	Validated	Rabbit	Upstate (Millipore)	06-195	https://www.millipore.com/catalogue/ item/06-195
PKC-pan_BetaII_pS660	PKC	Validated	Rabbit	CST	9371	http://www.cellsignal.com/products/9371.html
PRDX1	PRDX1	Under Evaluation	Rabbit	Sigma/ Atlas	HPA-007730	http://www.sigmaaldrich.com/catalog/product/ sigma/hpa007730?lang=en&region=US
Rab11	RAB11A RAB11B	Under Evaluation	Rabbit	CST	3539	http://www.cellsignal.com/products/3539.html

Raptor	RPTOR	Validated	Rabbit	CST	2280	http://www.cellsignal.com/products/2280.html
RBM15	RBM15	Validated	Rabbit	SDI/ Novus	21390002	http://www.novusbio.com/ RBM15-Antibody_21390002.html
Rictor	RICTOR	UsewithCaution	Rabbit	CST	2114	Discontinued, replaced by http://www.cellsignal.com/products/2280.html
Rictor_pT1135	RICTOR	Validated	Rabbit	CST	3806	http://www.cellsignal.com/products/3806.html
SCD1	SCD1	Validated	Mouse	Santa Cruz	sc-58420	http://www.scbt.com/ datasheet-58420-scd-cd-e10-antibody.html
SF2	SFRS1	Validated	Mouse	Invitrogen	32-4500	http://products.invitrogen.com/ivgn/product/ 324500?ICID=search-324500
TAZ	WWTR1	Validated	Rabbit	CST	2149	http://www.cellsignal.com/products/2149.html
Transglutaminase	TGM2	Validated	Mouse	Lab Vision	MS-224	https://thermo.dirxion.com/immunohistochemistry/ WebProject.asp?CodeId=7.6.3.2&BookCode=ihc12flx#
TFRC	TFRC	Validated	Rabbit	SDI/ Novus	22500002	http://www.novusbio.com/ Transferrin-Receptor-Antibody_22500002.html
TSC1	TSC1	Use with Caution	Rabbit	CST	4906	http://www.cellsignal.com/products/4906.html
Tuberin_pT1462	TSC2	Validated	Rabbit	CST	3617	http://www.cellsignal.com/products/3617.html
VHL	VHL	Use with Caution	Mouse	BD Pharmingen	556347	http://www.bdbiosciences.com/ ptProduct.jsp?prodId=12727&key=556347&param=search& mterms=true&from=dTable
XBP1	XBP1	Use with Caution	Goat	Santa Cruz	sc-32136	http://www.scbt.com/ datasheet-32136-xbp-1-r-14-antibody.html
TIGAR	C12ORF5	Under Evaluation	Rabbit	Epitomics	S1711	http://www.epitomics.com/products/product_info/ 3961/TIGAR-antibody-S1711.html
Acetyl-a-Tubulin (Lys40)		Under Evaluation	Rabbit	CST	5335	http://www.cellsignal.com/products/5335.html
p62 LCK ligand	SQSTM1	Under Evaluation	Mouse	BD Transduction Lab	610833	http://www.bdbiosciences.com/ ptProduct.jsp?prodId=32650&key=610833& param=search&mterms=true&from=dTable
ETS-1	ETS-1	Validated	Rabbit	BethYl	A303-501A	www.bethyl.com/product/A303-501A/ Ets-1_Antibody?referrer=search_default
Rab25	Rab 25	Validated	Rabbit	CST	4314	http://www.cellsignal.com/products/4314.html

Table S1: Information about the antibodies used for the RPPA measurements in this study.

Apoptosis	Cell Cycle	DNA Damage Response	EMT	Hormone_a
BAK, BAX, BID, BIM, CASPASE7-CLEAVEDD198, BADPS112, BCL2, BCLXL, CIAP	CDK1, CYCLINB1, CYCLIND1, CYCLINE1, CYCLINE2, P27PT157, P27PT198, PCNA	53BP1, ATM, BRCA2, CHK1PS345, CHK2PT68, KU80, MRE11, P53, RAD50, RAD51, XRCC1	FIBRONECTIN, NCADHERIN, COLLAGENVI, CLAUDIN7, ECADHERIN	ERALPHA, ERALPHAPS118, PR
**Hormone_b**	**PI3K/Akt**	**Ras/MAPK**	**RTK**	**TSC/mTOR**
AR, INPP4B, GATA3, BCL2	AKTPS473, AKTPT308, GSK3-ALPHABETA-PS21S9, GSK3PS9, P27PT157, P27PT198, PRAS40PT246, TUBERINPT1462, INPP4B, PTEN	ARAFPS299, CJUNPS73, CRAFPS338, JNKPT183Y185, MAPKPT202Y204, MEK1PS217S221, P38PT180Y182, P90RSK-PT359S363, SHCPY317, YB1PS102	EGFRPY1068, EGFRPY1173, HER2PY1248, HER3PY1298, SHCPY317, SRCPY416, SRCPY527	4EBP1PS65, 4EBP1PT37T46, 4EBP1PT70, P70S6KPT389, MTORPS2448, S6PS235S236, S6PS240S244, RICTORPT1135

Table S2: Proteins associated with the pre-defined pathways of interest.

6 Visual comparison of MixGlasso clustering to Akbani et al. and Hoadley et al.

In the main manuscript, we compare the clustering of the RPPA data using MixGlasso to the consensus clustering of different data types on material from an overlapping set of patients (Hoadley *et al.*, 2014), as well as the Pearson-Ward clustering done on the same RPPA data from the same set of patients as in our case study (Akbani *et al.*, 2014). Due to space constraints, we did not include a visual comparison in the main manuscript, but have provided one here in Figure S5. This figure is inspired by Figure 1 in Hoadley *et al.* (2014), and compares each individual cluster in our clustering (MixGlasso) against the Pearson-Ward clustering done on 181 proteins in Akbani *et al.* (2014) (PW-181), and the Pearson-Ward clustering done on 131 proteins (PW-131) as part of the input for the consensus clustering in Hoadley *et al.* (2014). The consensus clusting from Hoadley *et al.* (2014) is represented by the bar at the top (COCA). For the interpretation and discussion, see the main text.

References

Akbani, R., Ng, P. K. S., Werner, H. M. J., Shahmoradgoli, M., Zhang, F., Ju, Z., Liu, W., Yang, J.-Y., Yoshihara, K., Li, J., Ling, S., Seviour, E. G., Ram, P. T., Minna, J. D., Diao, L., Tong, P., Heymach, J. V., Hill, S. M., Dondelinger, F., Städler, N., Byers, L. A., Meric-Bernstam, F., Weinstein, J. N., Broom, B. M., Verhaak, R. G. W., Liang, H., Mukherjee, S., Lu, Y., and Mills, G. B. (2014). A pan-cancer proteomic perspective on the cancer genome atlas. *Nature Communications*, **5**.

Hoadley, K. A., Yau, C., Wolf, D. M., Cherniack, A. D., Tamborero, D., Ng, S., Leiserson, M. D., Niu, B., McLellan, M. D., Uzunangelov, V., *et al.* (2014). Multiplatform analysis of 12 cancer types reveals molecular classification within and across tissues of origin. *Cell*, **158**(4), 929–944.

Städler, N. and Mukherjee, S. (2013). Penalized estimation in high-dimensional hidden Markov models with state-specific graphical models. *Annals of Applied Statistics*, **7**, 2157–2179.
