# Supplementary material for: Molecular heterogeneity at the network level: high-dimensional testing, clustering and a TCGA case study
Source: Bioinformatics. 2017 May 23;33(18):2890–6. doi: 10.1093/bioinformatics/btx322 (PMC5590725; doi:10.1093/bioinformatics/btx322)
Supplement: Supplementary Figures [file btx322_suppl_stadler_et_al_si_figures.pdf]

Figure S1

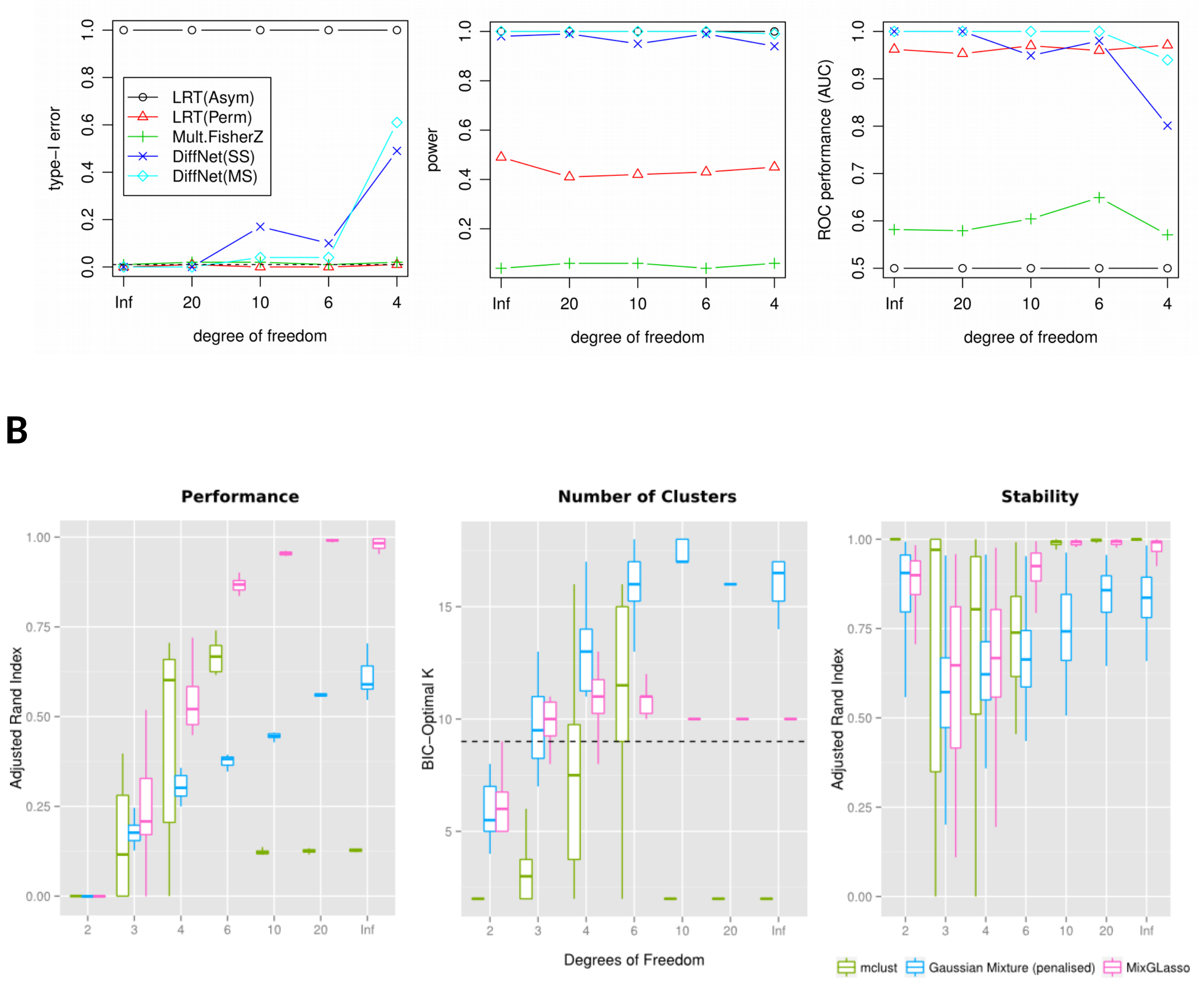

Fig S1. Simulation results under non-Gaussianity. Data were simulated from multivariate t-distributions with varying degrees of freedom (df; as df grows large, the Gaussian distribution is recovered). Sample sizes are identical to those in the TCGA data. (A) DiffNet performance. Leftmost plot shows Type-I error, middle plot show statistical power and right-most plot shows area under the ROC curve. [Results are based on 100 simulation runs. See Main Text for description of methods under comparison.] (B) Clustering performance. Left-most panel shows adjusted Rand index with respect to true cluster labels. The middle panel shows inferred number of clusters; the horizontal dashed line shows the true number of clusters. Right-most plot shows stability measure (obtained by subsampling each dataset 10 times and calculating the adjusted Rand index between the obtained clusterings). [Boxplots are over 10 datasets for each df value.]

Figure S2

A

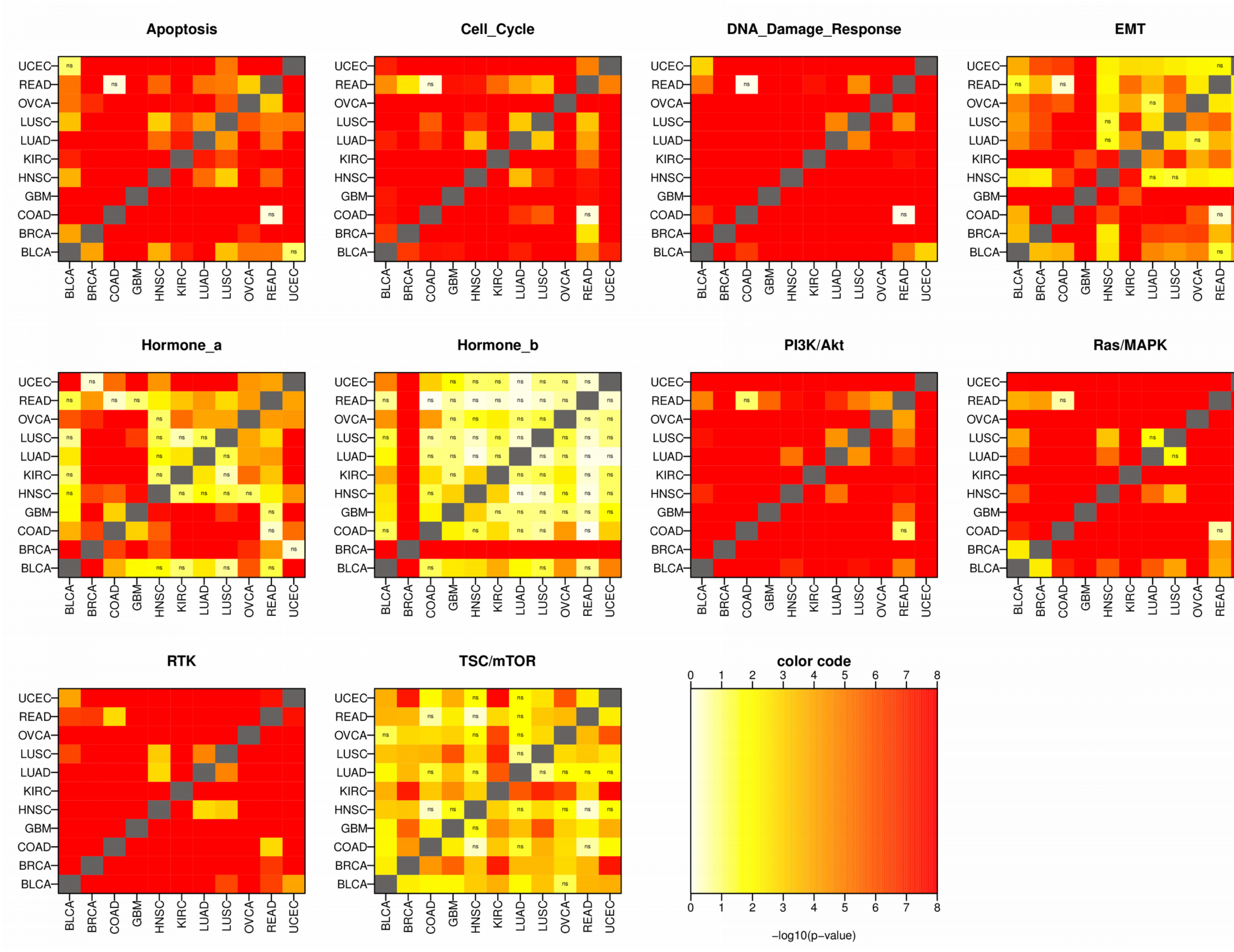
Apoptosis
Cell_Cycle
DNA_Damage_Response
EMT
Hormone_a
Hormone_b
PI3K/Akt
Ras/MAPK
RTK
TSC/mTOR
UCEC
READ
OVCA
LUSC
LUAD
KIRC
HNSC
GBM
COAD
BRCA
BLCA
ns
color code
0
1
2
3
4
5
6
7
8
−log10(p−value)

Figure S2

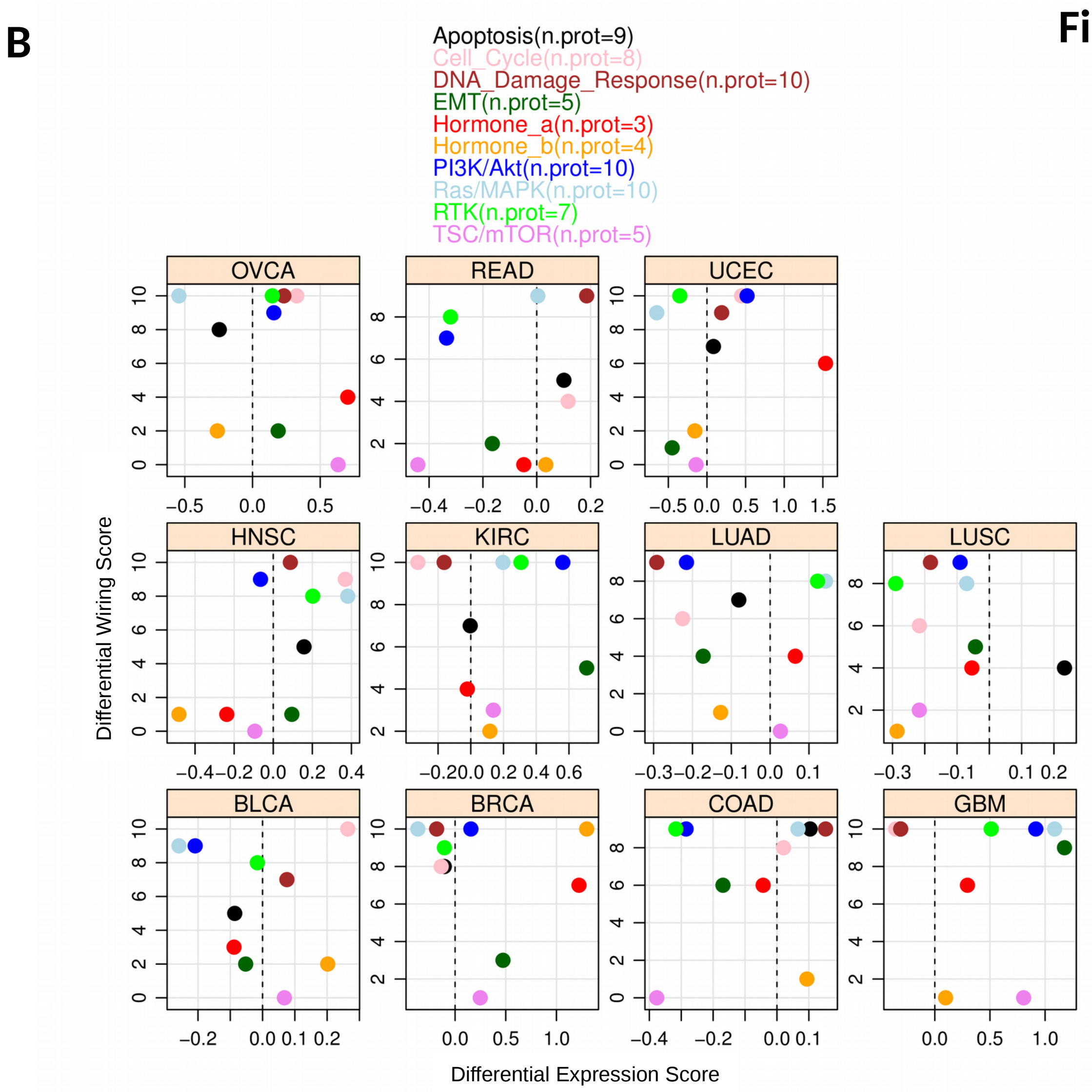

Fig S2. Statistical significance of differences between cancer-specific sub-networks. (A) The heatmap shows (FDR-corrected) p-values indicating significance of differences between cancer-specific sub-networks comprised of nodes belonging to specific pathways (“differential wiring”; see supplementary Table S2 for list of pathway members). To focus on network-related differences rather than differential expression, for each pair of cancer types, protein levels were normalized to have zero mean and unit variance as a pre-processing step. [“ns” indicates non-significant cancer pairs that have FDR corrected p-values greater than 1%.] (B) Scatter plots comparing differential scores from multivariate comparison of gene set members (“differential wiring”) with those from a classical gene set analysis (“differential expression”). See supplementary section S3 for a description of these scores. The colour of each dot indicates the pathway it represents.

Figure S3

A

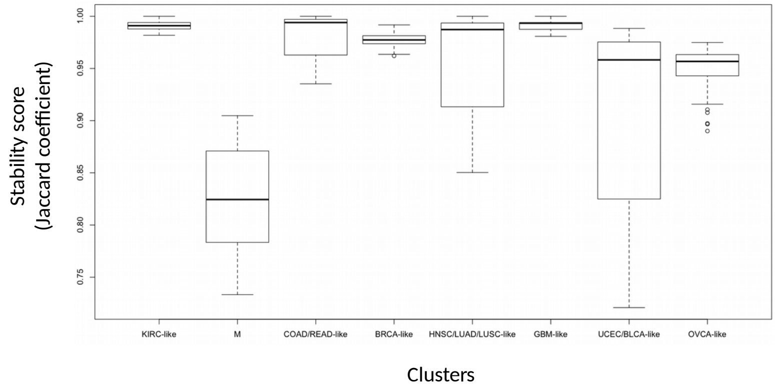

B

Diseases (rows) × Clusters (columns)

	OVCA-like	BRCA-like	UCEC/BLCA-like	HNSC/LUAD/LUSC-like	GBM-like	COAD/READ-like	“M”	KIRC-like	
BLCA	0	2	117	1	0	0	7	0	127
BRCA	70	631	29	3	0	6	8	0	747
COAD	0	1	1	0	0	325	7	0	334
GBM	0	0	0	1	204	0	10	0	215
HNSC	0	0	0	200	0	0	12	0	212
KIRC	0	0	2	0	0	0	16	436	454
LUAD	0	0	1	227	0	0	9	0	237
LUSC	0	0	0	191	0	0	4	0	195
OVCA	396	4	0	0	1	0	11	0	412
READ	0	0	0	1	0	127	2	0	130
UCEC	0	0	404	0	0	0	0	0	404
	466	638	554	624	205	458	86	436	

Clusters

Fig S3. (A) Stability of MixGlasso clustering when applied to the TCGA pan-cancer protein data. Cluster-wise stability scores were obtained following the resampling approach in Henning (2007). (B) Cross-tabulation of the *K*=8 identified clusters versus 11 TCGA cancer types.

Figure S4

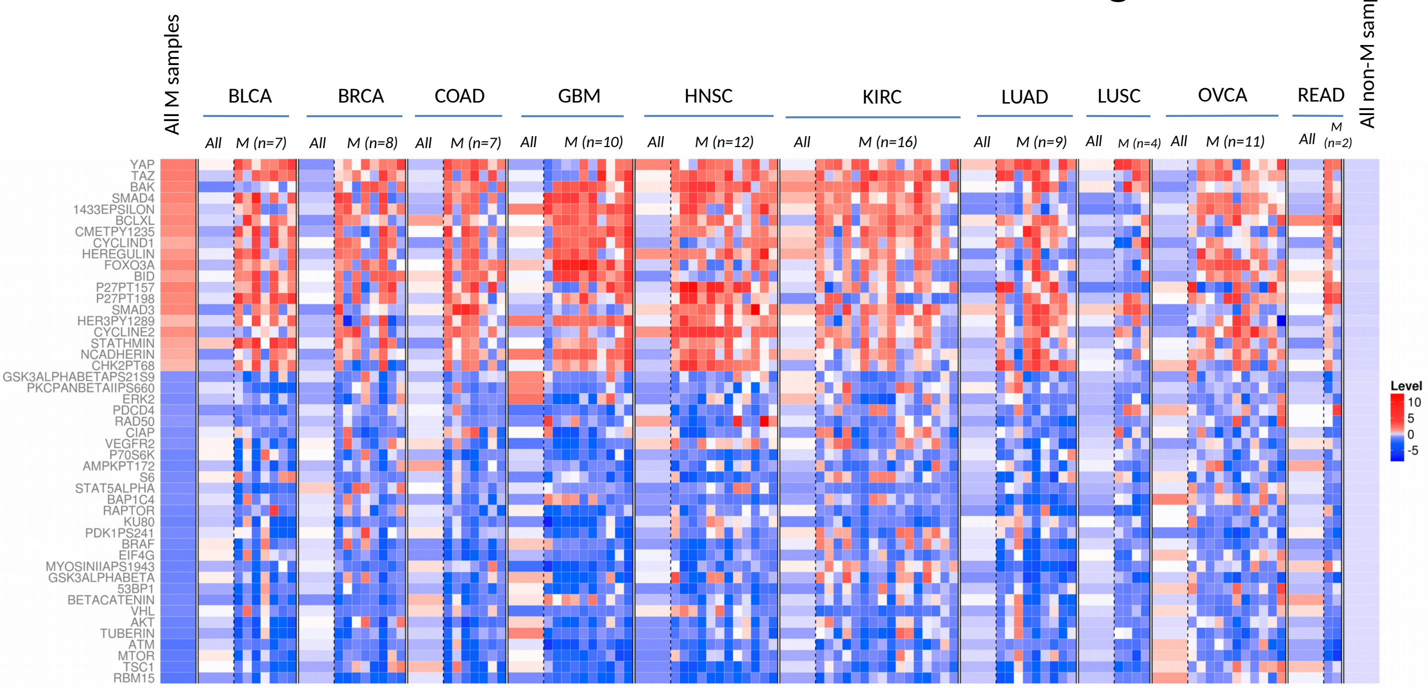

Fig S4. A novel protein-defined cluster that spans multiple cancers and lineages. Normalized protein levels for each of the n=86 samples assigned to the cluster M, arranged by cancer type (separated by double lines), shown alongside the cluster-specific profile for cluster M (``All M samples") and mean profiles for each cancer type (``All"). For example, below BRCA, ``M (n=8)" are the n=8 individual BRCA samples that appear in cluster M, while ``All" is the average profile over all BRCA samples. [Data were globally standardized such that each protein had zero mean and unit variance over all samples. Only proteins whose median level in cluster M is >0.75 standard deviations from the global mean for that protein are shown.]

Figure S5

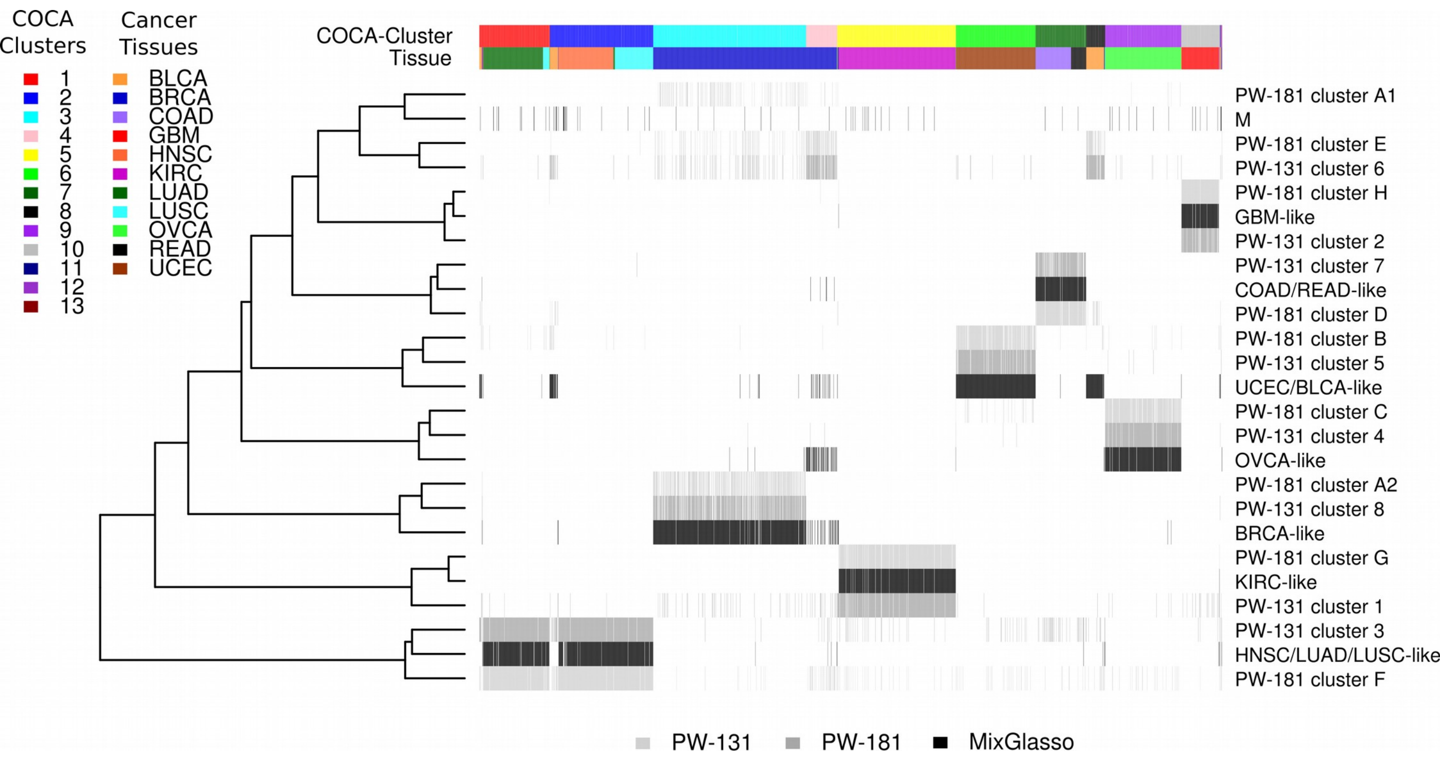

Fig S5. Comparison of MixGlasso clustering (in black), Pearson-Ward clustering on RPPA data from 181 proteins (PW-181, Akbani et al. 2014, in dark grey), Pearson-Ward clustering on RPPA data from 131 proteins (PW-131, Hoadley et al. 2014, in light grey) and consensus clustering of multiple data types (cluster of cluster assignments, COCA, Hoadley et al. 2014, coloured bar). Bars at the top show COCA clustering and cancer tissue labels. Rows represent individual clusters, labeled with the cluster name. Dendrogram shows hierarchical clustering of binary cluster assignments. The plot shows assignments on 2,809 samples that overlap between the publications and our case study.
